# Transformative 3D Printing of Carbon‐metal Nanocomposites as Catalytic Joule Heaters for Enhanced Ammonia Decomposition

**DOI:** 10.1002/advs.202413149

**Published:** 2025-04-27

**Authors:** Paul Smith, Jiachun Wu, Anthony Griffin, Kaleb Jones, Jeff Aguinaga, Ethan Bounds, Derek Patton, Yizhi Xiang, Zhe Qiang

**Affiliations:** ^1^ School of Polymer Science and Engineering University of Southern Mississippi Hattiesburg MS 39406 USA; ^2^ Dave C. Swalm School of Chemical Engineering Mississippi State University Mississippi State MS 39762 USA; ^3^ Department of Chemical and Biomedical Engineering University of Missouri Columbia MO 65211 USA

**Keywords:** additive manufacturing, decarbonization, hydrogen production

## Abstract

Electrified thermal chemical synthesis plays a critical role in reducing energy consumption and enabling the industrial decarbonization. While Joule heating offers a promising alternative to gas‐burning furnace systems by directly heating substrates via renewable energy supply, most approaches can only heat the reactor, not the catalytic sites. This limitation stems from the lack of methods to on‐demand create Joule heaters containing in situ loaded catalytic nanoparticles. This work introduces a scalable platform for producing carbonaceous Joule heaters embedded with catalytic nanoparticles from 3D‐printed polypropylene precursors, prepared through crosslinking, metal nitration immersion, and pyrolysis steps. Specifically, sulfonate groups on crosslinked PP can bind with metal ions, yielding well‐dispersed, nanosized particles within a carbon structure that maintains macroscopic dimensional accuracy throughout the manufacturing. The approach is modular, allowing control over particle size and composition. Structured carbon with in situ loaded nickel nanoparticles demonstrates efficient Joule heating, high catalytic activity, and significantly reduced activation energy for catalytic ammonia decomposition. This work provides an innovative material and manufacturing platform to produce structured, catalytically active Joule heaters for decarbonization of chemical synthesis and energy production.

## Graphical abstract

The work presents a scalable method for fabricating 3D carbon‐metal nanoparticle composites via additive manufacturing from low‐cost precursors, enabling their efficient use for catalytic Joule heating. This innovation enhances energy‐efficient chemical reactions, advancing decarbonized synthesis.

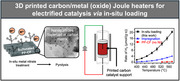

## 1 Introduction

The increasing consumption of fossil fuels for transportation and energy production has significantly elevated atmospheric CO₂ levels, leading to critical environmental and societal challenges. To address these issues, a paradigm shift toward a decarbonized society is urgently needed. Over the past decade, tremendous progress has been made across various sectors, including improving resource efficiency through recycling,^[^ ^1^ ^]^ developing renewable energy sources,^[^ ^2^, ^3^ ^]^ and advancing carbon capture technologies.^[^ ^4^, ^5^ ^]^ However, significant bottlenecks remain in the chemical and energy industries toward decarbonization, particularly in advancing hydrogen (H_2_) as a carbon‐free energy source and improving energy efficiency in heat generation for endothermic chemical reactions.^[^ ^6^ ^]^ Specifically, while hydrogen (H_2_) is poised to play a crucial role in the global energy portfolio, challenges with high‐density H₂ storage and transportation remain major hurdles to its widespread use.^[^ ^7^ ^]^ Ammonia (NH₃), containing 17.8 wt.% hydrogen, is a promising H₂ carrier due to its established infrastructure for long‐distance transportation and distribution. Therefore, NH_3_ decomposition is essential for enabling H₂ as a renewable energy source. Though various catalytic technologies including thermal, plasma, electro‐, and photo‐driven processes have been extensively explored to improve the reaction efficiency of NH₃ decomposition,^[^ ^8^, ^9^, ^10^ ^]^ thermal catalytic methods remain the most scalable. However, conventional thermal catalytic NH₃ decomposition faces challenges in distributed synthesis, especially associated with frequent dormancy, as significant heat waste is generated by the highly endothermic reaction. In 2022, ≈80% of the energy consumed in the U.S. chemical industry was dedicated to heat generation, contributing to ≈2 billion tons of CO₂ emissions.^[^ ^11^ ^]^

Joule heating is a pivotal technology for advancing decarbonized chemical and material synthesis, offering a pathway to electrify the heating process and generate thermal energy directly on a substrate.^[^ ^12^ ^]^ We note that conventional large‐scale heating processes in chemical reactions typically rely on gas furnaces which often produce non‐uniform temperatures inside the catalyst bed, leading to side reactions or reduced reaction efficiency.^[^ ^13^ ^]^ In contrast, Joule heating can supply thermal energy directly to the catalyst/reactants to minimize heat loss. This approach offers several key advantages, including accurate temperature control via energy input, rapid heating and cooling to extreme temperatures, reduced energy consumption due to superior heating efficiency compared to convection methods, and the ability to integrate with renewable power sources like solar and wind.^[^ ^14^ ^]^ In previous works, Joule heating‐enabled electrification has been demonstrated as a feasible alternative strategy for a variety of reactions including H_2_ production,^[^ ^15^, ^16^ ^]^ CO_2_ utilization,^[^ ^17^ ^]^ volatile organic compounds (VOCs) removal,^[^ ^18^ ^]^ and plastic recycling.^[^ ^19^ ^]^ For example, the use of Joule heaters for electrified methane steam reforming can lead to compact reactor designs, potentially 100 times smaller than current reformer platforms, which makes it highly desirable for distributed fuel and chemical production.^[^ ^20^ ^]^ It was also found that higher reaction conversions over Rh/γ‐Al_2_O_3_ wash‐coated silicon carbide foams can be achieved compared to oven‐heated setups.^[^ ^21^ ^]^ However, the production of H_2_ from NH_3_ decomposition through the Joule heating process is still underexplored. An example of such has been investigated by Badakhsh et al over a Joule‐heated nickel chromium aluminum (NiCrAl) foam impregnated with Ru nanoparticles.^[^ ^22^ ^]^ Moreover, Joule heating has been further established as an effective strategy for the rapid synthesis of catalytically active species. These approaches offer high energy efficiency, tunable particle sizes, and importantly serve to broaden the catalyst design space by enabling the high‐entropy mixing of immiscible metals.^[^ ^23^, ^24^, ^25^ ^]^

The widespread adoption of Joule heating in the chemical industry hinges on developing low‐cost, high‐performance heaters with control over properties and structures, that are capable of integrating catalytically active components.^[^ ^26^ ^]^ Additive manufacturing (AM) of carbonaceous materials has emerged as a promising tool for preparing high‐performance Joule heaters.^[^ ^27^ ^]^ These processes typically involve direct ink writing (DIW) of carbon‐based slurries followed by matrix removal,^[^ ^28^, ^29^, ^30^ ^]^ or direct pyrolysis of polymer precursors obtained through UV‐assisted printing^[^ ^31^, ^32^ ^]^ or fused filament fabrication (FFF) methods.^[^ ^33^ ^]^ Notably, recent advancements have demonstrated AM of carbon using a simple process and low‐cost polyolefin materials, which provides a promising and industrially relevant solution for producing carbon Joule heaters.^[^ ^34^, ^35^, ^36^ ^]^ However, their application in chemical synthesis has yet to be explored. Particularly, most chemical reactions rely on metal nanoparticles as catalysts, making the development of modular, catalytic nanoparticle‐embedded Joule heaters crucial for advancing decarbonization efforts. In most studies, catalysts are typically applied to the Joule heater via impregnation‐based and wash‐coating post‐treatment methods.^[^ ^14^ ^]^ The direct integration of catalytically active sites with Joule heating elements via a streamlined AM process, which could significantly enhance reaction outcomes through the electric current/field effect, remains untapped.

This study develops a scalable method for on‐demand fabrication of 3D‐structured carbon‐metal nanoparticle composites, derived from commodity 3D‐printed polypropylene (PP) filaments, with exceptional dimensional accuracy, exhibiting less than 4% dimensional shrinkage when converting the plastic precursor to the final carbonized part. Metal precursors are introduced into crosslinked PP parts through immersion in a metal nitrate solution, which, upon pyrolysis, forms nanoparticles within the carbon framework; this approach enables the integration of metal nanoparticles with modular chemical identities and loading content into a carbonaceous matrix, offering high design flexibility and structural adaptability. The resulting Joule heaters can be directly employed in various catalytic applications, where this work specifically demonstrates their use in NH₃ decomposition reactions. The embedded nanoparticles exhibit superior catalytic performance compared to conventional impregnation counterparts, while significantly reducing energy consumption due to their highly efficient Joule heating performance. Our transformative AM of carbon‐metal nanoparticle composites is a versatile process, enabling the creation of modular catalytic Joule heaters that enhance reaction performance in electrified chemical synthesis. This approach holds significant potential for advancing a sustainable future by facilitating the development of energy‐efficient, distributed chemical and energy sectors.

## 2 Results and Discussion

**Figure 1:**
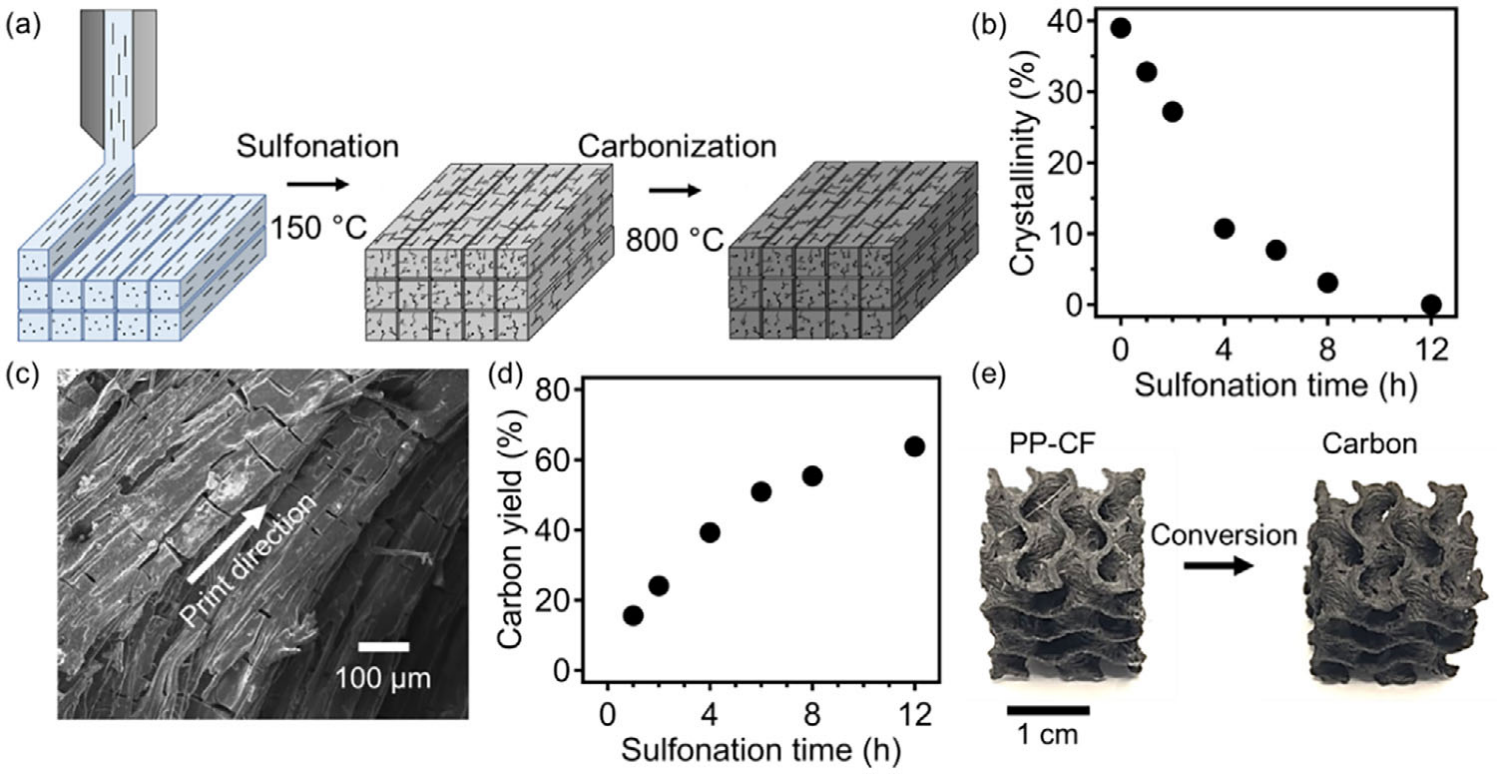
a) A schematic demonstration of AM of carbon process, including steps of FFF printing sulfonation‐induced crosslinking, and subsequent carbonization. b) Crystallinity change of model PP‐CF specimens as a function of sulfonation time. c) SEM image of carbon morphology from PP‐CF precursors. d) Carbon yield of PP‐CF model system as a function of sulfonation time. e) Photos showing the conversion of printed PP‐CF to carbon parts.

A typical procedure to prepare 3D printed carbon materials is illustrated in **Figure** **1**a, using PP‐CF (polypropylene containing 15 wt.% chopped carbon fiber) as the starting material, in conjunction with sulfonation‐induced cross‐linking and carbonization steps. To provide a brief description, a fused filament fabrication (FFF)‐based method was first used to generate precursor structures which were crosslinked at 150 °C, and subsequently pyrolyzed under a nitrogen atmosphere to form structured carbon. A key metric indicating the progress of the crosslinking reaction is the associated change in PP crystallinity as a function of reaction time. As shown in Figure 1b, an initial PP crystallinity of ≈38% was observed and reduced upon the progression of crosslinking, leading to a degree of crystallinity of ≈12% after 4 h and 0% after 12 h, indicating the amorphous nature of crosslinked PP resulting from sulfonation.^[^ ^37^ ^]^ For the rest of this study, a sulfonation time of 12 h was used to cross‐link 3D‐printed PP‐CF parts. Figure S1 (Supporting Information) shows the scanning electron microscopy (SEM) image of PP‐CF after cross‐linking for 12 h, where the presence of cracks is associated with the mechanical stress produced during the diffusion‐controlled sulfonation of PP‐CF. These cracks propagated throughout the sample, facilitating the diffusion of sulfuric acid, thus enabling crosslinking throughout the entire sample. This cracking phenomenon originates from the mismatch in volumetric changes between the hydrophilic crosslinked domains in the shell of the printed filament and the pristine PP regions in the core. Subsequently, these micron‐sized cracks can propagate throughout the printed part, which is a complex process influenced by various factors, such as reaction kinetics, acid diffusion kinetics, critical printed polymer filament dimensions (diameter), and CF filler orientation. The resulting cracked morphology was maintained upon carbonization, as presented in Figure 1c; in this fiber‐containing system, cracks were found in both parallel and perpendicular to the printing direction.

Once crosslinked, PP‐CF can act as an efficient carbon precursor. Figure 1d shows the carbon yield of PP‐CF as a function of sulfonation time, which can achieve at least 65 wt.% upon sulfonation for 12 h. Notably, carbon derived from polyolefins is often microporous in nature due to the ejection of small molecules during pyrolysis and contains a largely amorphous structure (non‐graphitizable) when pyrolyzed to 800 °C.^[^ ^38^, ^39^, ^40^ ^]^ Of particular importance, our samples can exhibit very low shrinkage upon the pyrolytic conversion of printed parts to carbons. As shown in Figure 1e, as‐printed and carbonized specimens were nearly identical in shape and size with a dimensional shrinkage in the in‐plane direction of ≈2% and a dimensional shrinkage in the out‐of‐plane direction of ≈5% upon pyrolysis (diagram indicating these directions is provided in Figure S2, Supporting Information). We note that during FFF printing of PP‐CF model structures, small deviations in dimensional accuracy were observed between the 3D models and printed parts were observed leading to an increase in the size of ≈2% in the in‐plane dimensions and ≈1% in the out‐of‐plane dimension. These changes may be attributed to polymer flow during printing and small errors in the out‐of‐plane step building up over time. The excellent structural retention behavior is attributed to the presence of CF hindering the shrinkage/densification of the polymer matrix upon carbonization, which provides an important advantage for enabling accurate AM of carbon‐based materials.

**Figure 2:**
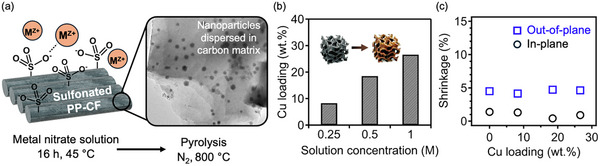
a) Schematic representation of carbon‐metal nanocomposite additive manufacturing wherein sulfonated PP‐CF structures are submerged in solutions of water/ethanol mixtures containing metal nitrate salts, followed by direct pyrolysis. b) Weight percent of copper in 3D printed composite materials with increasing concentration of metal nitrate precursor solution. c) Dimensional shrinkage from printed specimen to carbon‐copper nanocomposites with increasing copper loading level. Square markers represent out‐of‐plane shrinkage, and round markers represent in‐plane shrinkage.

This work presents an innovative and generalizable method for direct incorporation of metal nanoparticles into the 3D printed carbon framework, which is achieved by submerging cross‐linked PP‐CF into a metal nitrate solution, allowing the diffusion of metal precursors into the printed parts. Specifically, fully cross‐linked PP‐CF samples were submerged in a solution composed of a metal nitrate salt dissolved in a 50:50 volumetric mixture of ethanol and deionized water for 16 h at 45 °C. Upon pyrolysis at high temperatures, the precursor is converted to carbon, while the metal nitrates are transformed into metal particles. As shown in **Figure** **2**a, the crosslinked PP‐CF is typically enriched with a substantial amount of sulfonic acid groups on the polymer backbones. These functional groups not only greatly enhance the hydrophilic nature of 3D printed parts but also create binding sites between the polymer and metal ion species. This mechanism is important to highlight as it differs from the seminal work by Greer and co‐workers, where structured metal oxide was formed through the infusion of metal nitrates into 3D‐printed hydrogel templates.^[^ ^41^ ^]^ In their study, the metal precursors were uniformly distributed within the polymer matrix, resulting in the formation of a continuous metal framework upon calcination. However, in our work, these acid group sites enable the synthesis of nanosized particles after carbonization, similar to other studies that employ metal precursors for the functionalization of polymeric materials with in situ synthesized nanoparticles.^[^ ^42^, ^43^, ^44^, ^45^ ^]^ In our approach, the concentration of metal nitrate in the solution offers a facile handle to control the loading content of metal particles in the resulting carbon‐matrix metal nanocomposites.

This study first employed copper metal as a model system using copper(II) nitrate hemipentahydrate precursors. Favored interactions between metal ions and sulfonated PP were observed via X‐ray photoelectron spectroscopy (XPS) analysis where the bonding state of Cu was shown to differ between the nitrate salt and sulfonated PP‐CF treated with the copper solution. Corresponding *Cu 2p* spectra of the copper(II) nitrate hemipentahydrate and sulfonated PP‐CF treated with the copper solution are provided in Figure S3 (Supporting Information) where a distinct shift is observed from a *Cu^2+^* state (at 935.2 eV) to a *Cu^+^/Cu^0^* state (at 932.6 eV) upon exposure to the sulfonated PP‐CF.^[^ ^46^ ^]^ This result suggests that the copper ions can bind with negatively charged SO_x_ groups on the sulfonated PP backbones. Figure 2b illustrates the loading level of copper compounds in the model carbon‐metal nanocomposites, as determined by thermogravimetric analysis (TGA) measurements, where the carbon was completely thermally decomposed under air; a representative TGA curve is provided in Figure S4 (Supporting Information). When a 0.25 m solution of copper(II) nitrate hemipentahydrate was used, a loading level of ≈8 wt.% of copper in the carbon composites was observed. Increasing the metal nitrate solution concentration to 1 m led to a higher loading level of copper nanoparticles reaching ≈27 wt.%. The effects of copper loading are further reflected in the increase in the mass of these samples after pyrolysis, as shown in Table S1 (Supporting Information), which corresponds closely to the values reflected in TGA results. As shown in the inset of Figure 2b, the high loading level of copper nanoparticles resulted in a change in the visual appearance of these 3D printed samples after carbonization, from the typical black color of carbon to the ruddy brown color characteristic of copper metal. As previously discussed, a principal advantage of using a fiber‐filled carbon precursor system (i.e., PP‐CF precursor) is its ability to enable very minimal shrinkage from printed to carbonized parts, providing control over manufacturing accuracy. The potential impact of metal precursor loading content on the shrinkage behaviors of these copper nanoparticle‐containing samples was assessed, as shown in Figure 2c. It was found that there were no notable deviations in shrinkage with increasing metal loading content, as all remained to be ≈2% along in‐plane direction and ≈5% along out‐of‐plane direction. The anisotropic shrinkage behavior observed in this system is the result of the inclusion of CFs within the PP precursor filament which are largely aligned in the in‐plane printing direction. During pyrolysis, the presence of CF fibers can restrict shrinkage and densification of the PP matrix which is consistent with a previous study.^[^ ^35^ ^]^ Figure 2c suggests that the presence of CF in our system dictates shrinkage behaviors regardless of the loading level of metal oxide within the 3D printed structures, providing the ability to on‐demand produce carbon‐metal nanocomposite materials with high design flexibility and dimensional accuracy.

**Figure 3:**
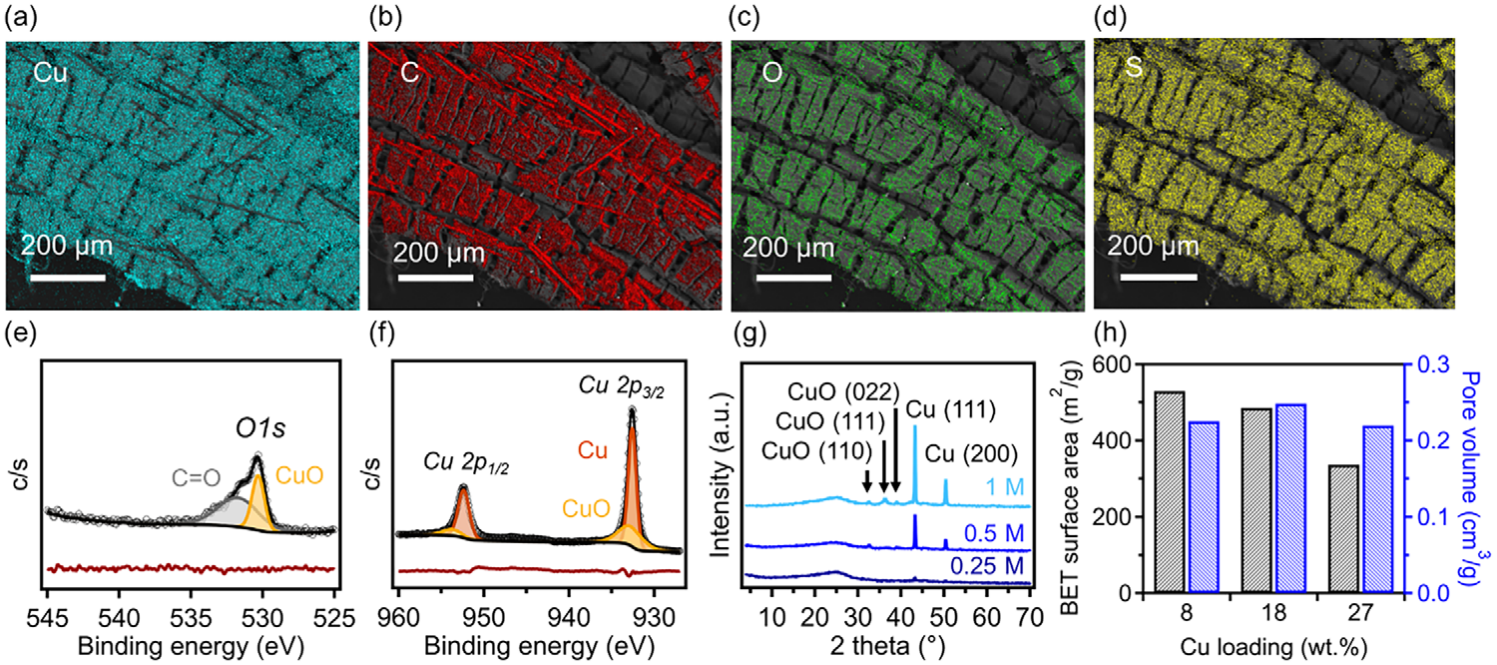
SEM EDX elemental mapping images of 3D printed carbon‐copper nanocomposites, including a) copper, b) carbon, c) oxygen, d) sulfur. e) XPS high‐resolution spectrum of *O 1s* with fitting (black) and residuals (shown beneath the spectrum in dark red). f) XPS high resolution of *Cu 2p* with fitting (black) and residuals (shown beneath the spectrum in dark red). g) XRD spectra of carbon‐copper nanocomposites as a function of solution molarity of metal nitrate precursors. h) BET surface area and pore volume of 3D printed carbon‐copper nanocomposites as a function of Cu loading content, corresponding to molarities presented in (g).

To understand the chemical composition of these 3D printed composites, energy dispersive X‐ray (EDX) spectroscopy was performed. SEM images with elemental mapping, as shown in **Figure** **3**a–d, reveal that copper, carbon, oxygen, and sulfur are uniformly distributed across the sample at a macroscopic scale. Specifically, the sample prepared using a 1 m copper(II) nitrate hemipentahydrate solution was found to contain ≈64 wt.% carbon and 32 wt.% copper, which quantitatively agrees with our TGA results. Very low amounts of oxygen (≈3 wt.%) and sulfur (≈1 wt.%) were also identified, which can be associated with the sulfonation reaction for crosslinking of PP‐CF samples. Moreover, XPS was performed on these nanocomposite samples. The XPS survey scan, presented in Figure S5 (Supporting Information), identified the presence of carbon (≈80 at%), copper (≈10 at%), oxygen (≈5 at%), and sulfur (≈1.3 at%); the copper content from XPS measurement corresponded to ≈36 wt.%, which is slightly higher than the values obtained from TGA measurements of bulk samples; this difference may originate from the fact that TGA provides a bulk averaged result, while XPS focuses specifically on the sample surface. High‐resolution XPS scan of *O 1s* (Figure 3e) revealed a broad peak centered ≈531.5 eV, and its deconvolution indicates the presence of two different bonding types, including *C = O* type at 531.7 eV (≈52.5 at%) and *Cu‐O* type (≈47.5 at%) at 530.3 eV. We note that *C = O* bonds are observed in our control samples with the absence of metal nanoparticles, which suggests that they are likely associated with oxygen heteroatoms in the carbon framework derived from sulfonated PP precursor. Furthermore, as shown in Figure 3f, the carbon‐copper nanocomposite exhibits a major peak corresponding to the *Cu 2p_3/2_* region, which can be deconvoluted into a doublet at 932.6 and 933.1 eV. A similar phenomenon is observed in the *Cu 2p_1/2_* region, with two peaks at 952.3 and 954.5 eV, respectively. These XPS results suggest that copper in our sample is present primarily in the elemental state, with a portion in oxidized states. The oxidation is likely due to surface oxidation occurring during sample storage when exposed to air.^[^ ^47^, ^48^, ^49^ ^]^ These findings can be further corroborated by the X‐ray diffraction (XRD) spectroscopy results shown in Figure 3g. XRD peaks corresponding to both elemental copper and copper oxide can be identified, and their intensity increases with a higher molarity of metal nitrate precursors; an XRD spectra of carbon derived from PP‐CF without treatment is provided in Figure S6 (Supporting Information). Specifically, in Figure 3g two peaks at 2θ values of 43.3° and 50.4° correspond to (111) and (200) planes of metallic copper, respectively.^[^ ^50^ ^]^ Peaks associated with the presence of copper(II) oxide were also observed at 2θ values of 32.4°, 35.9°, and 39.2°, corresponding to (110), (110), and (022) indices, respectively, albeit with much lower intensities (due to sample oxidation under ambient conditions).^[^ ^51^, ^52^ ^]^ XRD spectra of all PP‐CF derived samples exhibit a broad peak at a 2θ value of ≈24.3°.

Notably, a previous study indicated that 3D‐printed carbon from PP‐CF precursors can exhibit varied pore textures depending on the crosslinking conditions.^[^ ^35^ ^]^ In this work, while the crosslinking time was consistent at 12 h, it is important to understand the impact of copper loading on the pore size and surface area of the resulting 3D‐printed parts. Liquid nitrogen physisorption measurements were performed to determine the surface area and pore characteristics of these carbon‐copper nanocomposites. In the PP‐CF derived carbon system with the absence of metal nanoparticles, its BET surface area can achieve 318 m^2^ g^−1^ (Figure S7, Supporting Information). As copper is introduced into the system (Figure 3h), a marked increase was first observed at low loading levels where a surface area of 529 m^2^ g^−1^ was observed with a copper loading level of 8 wt.%. This increase in surface area is likely the result of the activation of carbon framework from the presence of nitrate compounds during pyrolysis; a similar increase was observed in previous studies when melamine was present in carbonizing polymeric precursors.^[^ ^53^, ^54^ ^]^ However, as the copper loading content increased further, a reduction in the surface area of the carbon‐copper nanocomposite samples was observed, decreasing from 485 m^2^ g^−1^ with 18 wt.% Cu to 336 m^2^ g^−1^ with 27 wt.% Cu. This reduction is anticipated, as the increased mass loading of Cu in the final composites leads to structures with a higher density and may be also attributed to the occupying of pores with metal species.^[^ ^55^, ^56^ ^]^

**Figure 4:**
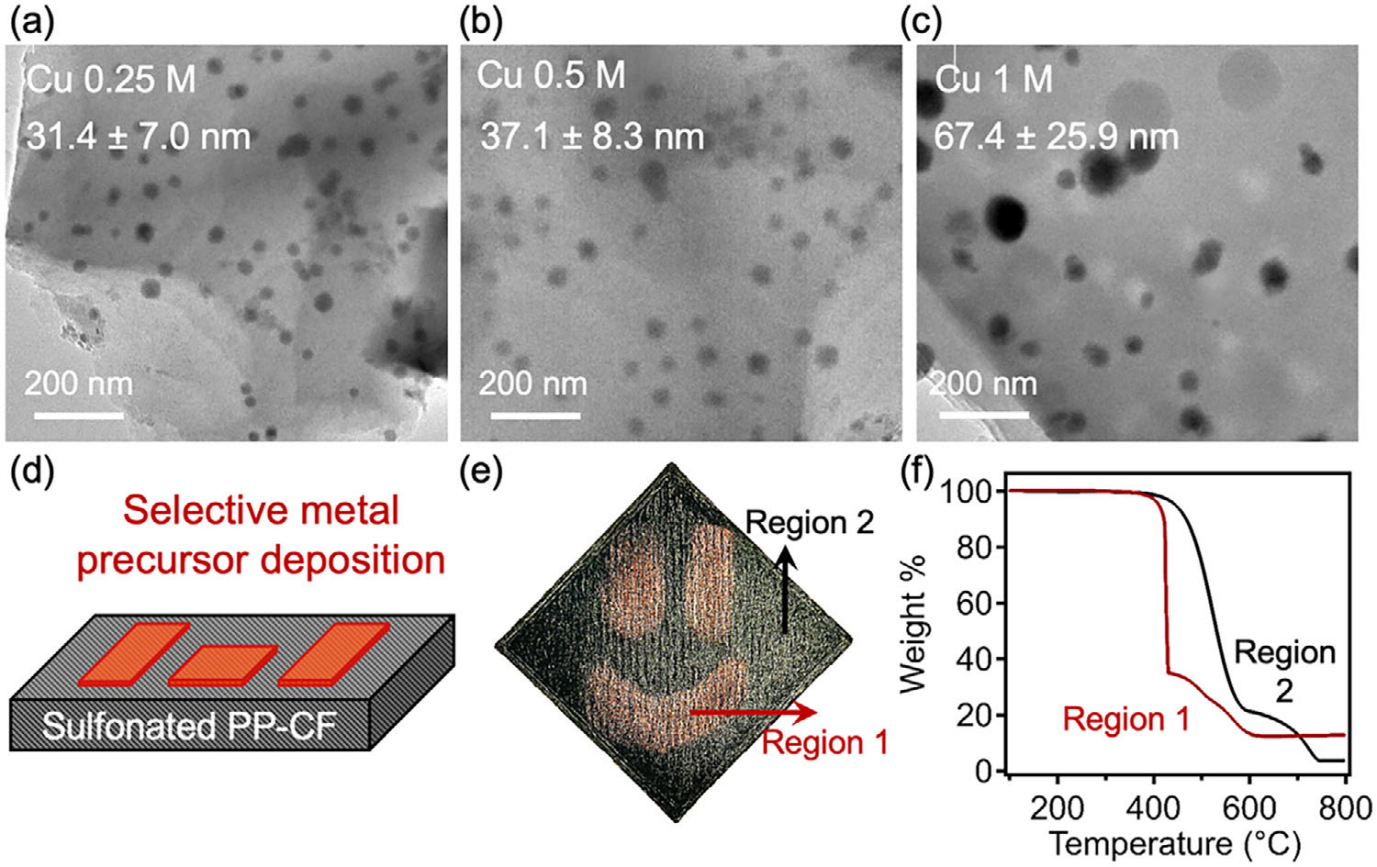
a–c) TEM micrographs of carbon‐copper nanocomposites derived from 0.25, 0.5, and 1 m metal nitrate precursor solutions, and averaged particle sizes labeled in the figure are provided based on over 30 measurements. d) Schematic of selective application method. e) Demonstrator part resulting from selective application of metal solution (sample size: 5 cm x 5 cm) and f) representative TGA curves for treated (red) and untreated (black) portions of this sample heated at 1 °C min^−1^ under air.

TEM was performed to gain further insight into the morphology of copper particles within these nanocomposites. As shown in **Figure** **4**a–c, copper nanoparticles presenting as dark regions in the images were found to form spherical shapes, which were well‐dispersed within the matrix of the carbon. Furthermore, as the concentration of metal precursor solution increased, the average size of these particles was found to increase. Specifically, using a copper nitrate solution with a concentration of 0.25 m, an average copper particle size of 31.4 ± 7.0 nm was observed in carbonized parts. As the solution concentration increased to 1 m, the average particle size grew to 67.4 ± 25.9 nm. Particle size distributions for each concentration are provided in Figure S8 (Supporting Information) where increased variably in nanoparticle size was observed when a higher concentration metal nitrate precursor solution was used. These results suggest that varying the molarity of the treatment solution not only alters the overall metal loading content but also influences the size of the resulting nanoparticles. Specifically, the 1 m nitrate concentration results in noticeably greater size variation of copper nanoparticles, which may require further optimization, such as via refining the nitrate precursor infiltration time or potentially changing the choice of metal precursors, to achieve more uniform size distributions within the carbon framework.

To further exhibit the utility of this approach, a demonstration structure was produced. This structure was created by applying a 0.5 m copper nitrate solution to a printed and sulfonated PP‐CF substrate prior to pyrolysis using a simple method schematically represented in Figure 4d. The resulting structure (a smiley face) is presented in Figure 4e. Two regions were identified: Region 1 where copper nitrate solution was applied, displaying the ruddy brown color of copper, and Region 2 where no solution was applied which appears as black. Representative TGA results of the distinct regions, including PP‐CF derived carbon (black) and the copper‐loaded sections of the smiley face (red) are shown in Figure 4f. The distinct plateau at ≈17 wt.% in the copper‐loaded sample is consistent with previous results, indicating that our method can achieve spatially controlled deposition of nanoparticles within the structured carbons.

**Figure 5:**
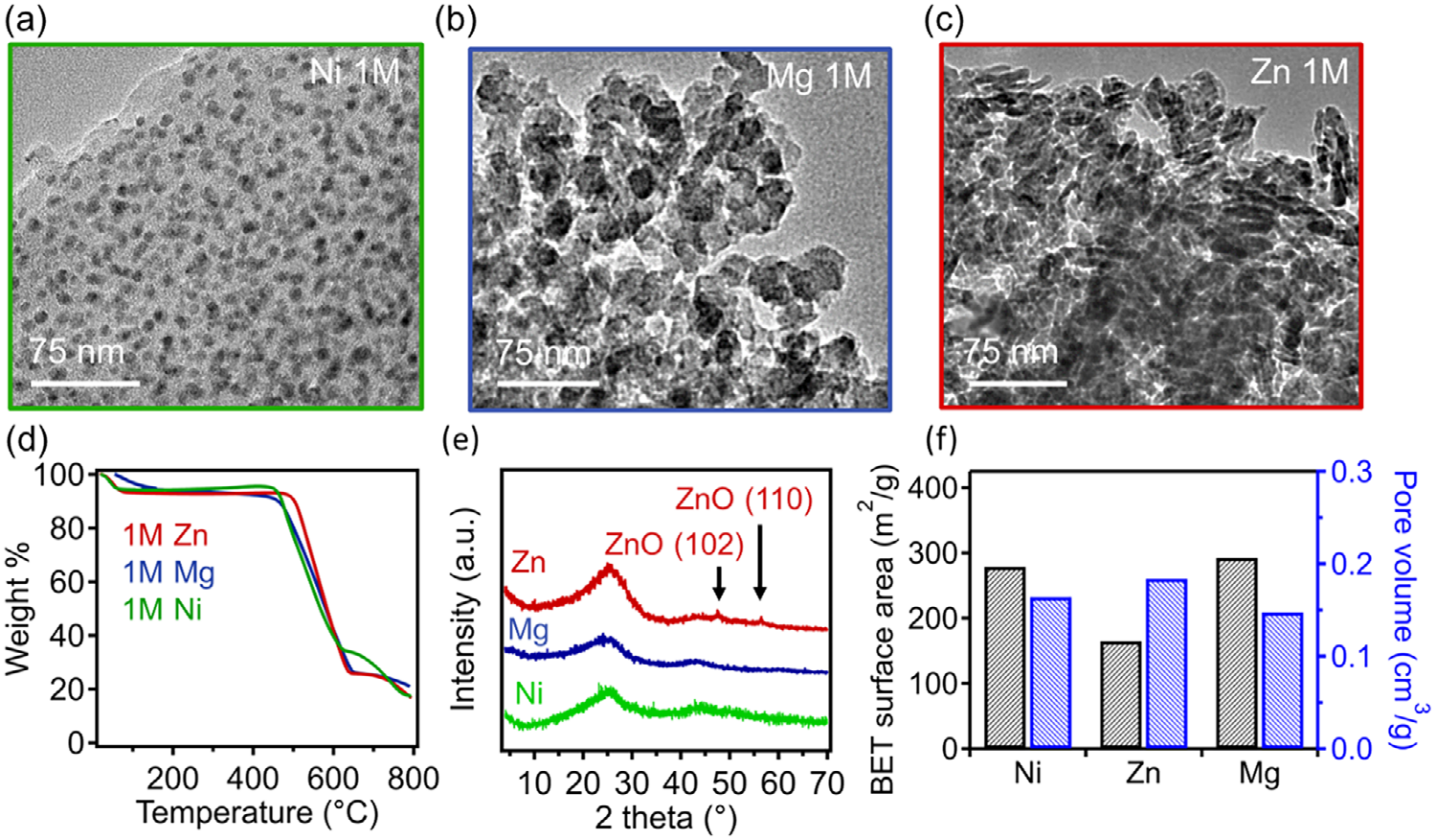
TEM images of carbon‐metal nanocomposites derived from 1 m metal nitrate solutions of: a) nickel (Ni), b) magnesium (Mg), and c) zinc (Zn). d) shows their TGA profiles corresponding to these samples. e) XRD spectra of these samples. f) BET surface area and pore volume as a function of metal identity.

A key advantage of our method for preparing 3D‐printed carbon‐metal nanocomposites lies in its modularity and versatility in tailoring the chemical composition of the final systems, which can be enabled by using various metal nitrate salts, allowing for a broad range of material selection. By adjusting the metal content and identity, we can alter and optimize the properties of the composites, offering system tunability to meet the demands of specific applications. To demonstrate this advantage and examine how changing the material identity of the metal precursor influences the structure and properties of resulting 3D printed nanocomposites, a series of samples were prepared using hydrated nitrate salts of nickel (Ni), zinc (Zn), and magnesium (Mg). These composites were manufactured using a 1 m solution of each metal salt, following the previously discussed methodology. The resulting SEM EDX maps of the corresponding metals in the final carbon‐metal nanocomposites are all included in Figure S9 (Supporting Information). The nanostructures of these samples were investigated via TEM as presented in **Figure** **5**a–c. Despite the same molar concentration used, samples containing Ni (Figure 5a) displayed smaller nanoparticles with more uniform size distributions than what was observed with Cu, exhibiting an average particle diameter of 7.2 ± 1.6 nm. This result suggests that the identity of the metal and the selection of precursors can have a significant impact on the resulting particle size and uniformity. Moreover, 3D‐printed nanocomposites containing both Zn and Mg exhibit larger‐size agglomerates. Specifically, Mg formed largely sphere‐like agglomerates, whereas Zn formed aggregates composed of needle‐like structures (Figure S10, Supporting Information). These behaviors have been observed previously during the formation of metal oxide nanoparticles from Mg‐based^[^ ^57^, ^58^ ^]^ and Zn‐based nitrates,^[^ ^59^ ^]^ which can be attributed to their high surface energies leading to a tendency toward aggregation.^[^ ^42^ ^]^ TGA was performed under air for these samples and their thermograms are provided in Figure 5d, which indicated the presence of Ni particles at 18 wt.%, Zn particles at 17 wt.%, and Mg particles at 21 wt.% loading content. These results confirm that our approach is broadly applicable for introducing a variety of metal nanoparticles into 3D‐printed carbon structures, achieving high and consistent loading levels.

Further study of the elemental composition and bonding state of these nanocomposites via XPS measurements is shown in Figures S11–S13 (Supporting Information). In the Ni system, survey scans (Figure S11a, Supporting Information) revealed the presence of Ni species at ≈4.5 at% (corresponding to 18 wt.%). The high‐resolution scan of the *Ni 2p* region, as shown in Figure S11b (Supporting Information), revealed these species were likely predominantly nickel oxide with a very low amount of hydroxide. This was evidenced by a characteristic doublet in the *Ni 2p_3/2_* peak with subpeaks located at 853.7 and 855.6 eV, corresponding to the contributions of NiO and Ni(OH)_2_, respectively.^[^ ^60^, ^61^ ^]^ While the presence of carbon‐oxygen bonding in the *O1s* region (Figure S11c, Supporting Information) makes quantitative peak deconvolution difficult in this system, a clear peak associated with *Ni‐O* bonding was observed at 529.3 eV. Similarly, a survey scan of carbon‐magnesium nanocomposite (Figure S12a,Supporting Information) indicated the content of Mg species at 10.4 at% (corresponding to 17 wt.%). High‐resolution scans of the *Mg 1s* region (Figure S12b,Supporting Information) revealed a single peak centered at 1304.3 eV, corresponding well to the formation of MgO and was further supported by a peak present in the *O1s* scan (Figure S12c, Supporting Information) at 529.6 eV associated with the *Mg‐O* bond.^[^ ^62^ ^]^ Carbon nanocomposites produced using the Zn precursor solution were found to contain ≈8.1 at% Zn (29 wt.%) from the XPS survey scan, as shown in Figure S13a (Supporting Information). Analysis of the *Zn 2p* region (Figure S13b, Supporting Information) revealed the presence of a sharp *Zn 2p_3/2_* peak at 1020.8 eV, indicating the primary presence of metallic Zn. However, a broad peak in the *O1s* scan (Figure S13c, Supporting Information) with features located ≈530.4 eV indicated the presence of Zn‐O bonding.^[^ ^63^ ^]^ Further study of the carbon‐zinc nanocomposites via XRD revealed peaks at 2θ values of 47.5° and 56.3°, corresponding to the (102) and (110) indices of zinc(II) oxide.^[^ ^64^ ^]^ In contrast, the samples containing Mg and Ni nanoparticles remained largely amorphous. Figure 5f shows that Ni‐ and Mg‐containing carbon samples exhibited similar surface areas, just below 300 m^2^ g^−1^, while Zn‐containing carbon samples showed a lower surface area of 170 m^2^ g^−1^. The pore volumes of these samples also varied, ranging from 0.15 to 0.18 cm^3^ g^−1^. These findings indicate that the identity of metal precursor significantly influences nanoparticle morphology, crystallinity, carbon surface area, and pore volume, which may be due to variations in metal formation mechanisms from nitrate precursors and their distinct thermal degradation profiles. Due to the wide variety of hydrous metal salts commercially available, this technique may be applicable to various catalytic nanoparticles, such as oxides of iron, cobalt, zirconium, manganese, and many others.

**Figure 6:**
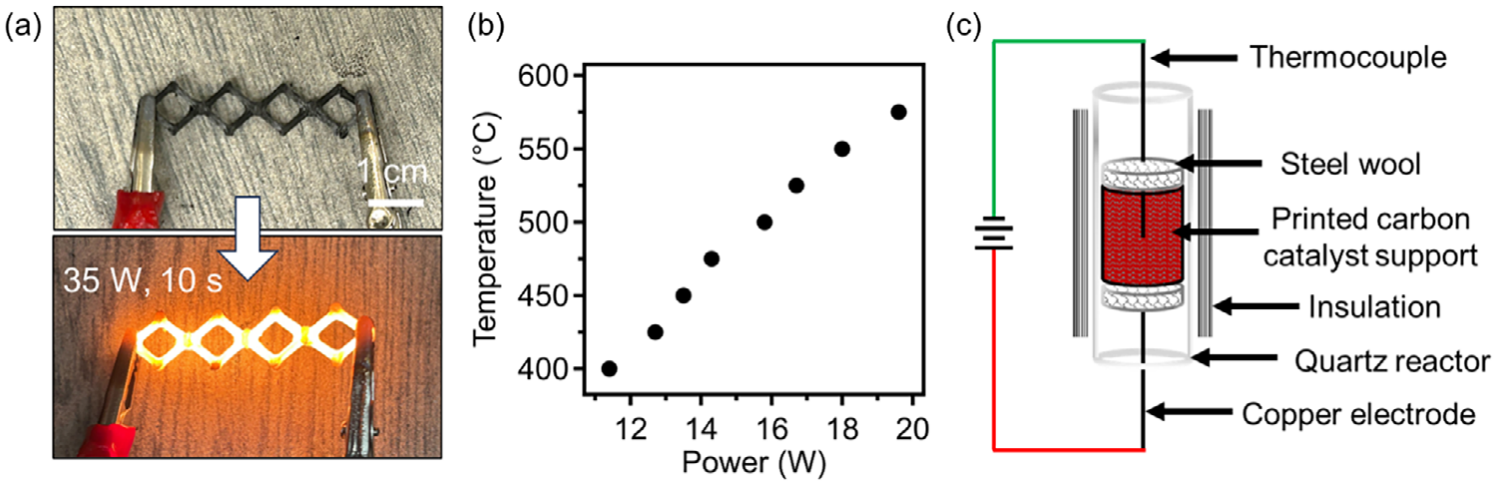
a) Joule heating performance of PP‐CF derived carbons reaching a glowing state after 10s of exposure to 35 W. b) Temperature versus power relationship for PP‐CF derived catalyst support containing 5 wt.% Ni nanoparticles. c) Schematic of reactor design used to measure the catalytic performance of these materials.

The ability to achieve a uniform distribution of nanosized Ni nanoparticles within 3D‐printed carbon structures is particularly advantageous for catalytic applications, which can potentially lead to consistent and efficient catalytic activity while maximizing the surface area available for reactions. Additionally, the integration of these well‐dispersed nanoparticles within a robust carbon framework enhances the durability and stability of the catalyst, making it highly effective for a wide range of industrial and environmental processes. Specifically, Ni‐based heterogeneous catalysts have been extensively studied for various reactions, such as NH_3_ decomposition (for H_2_ production),^[^ ^8^, ^65^ ^]^ biomass transformation,^[^ ^66^ ^]^ and CO_2_ conversion.^[^ ^67^ ^]^ An important feature of PP‐CF derived carbon is its ability to act as a highly efficient heating element in Joule heating applications; this property is demonstrated in **Figure** **6**a, where a printed carbon structure derived from PP‐CF emitted an orange‐white glow after exposure to 35 W for just ten seconds, suggesting heating to very high temperatures (>1000 °C).^[^ ^68^ ^]^ This excellent Joule heating performance makes these carbons very promising candidates for applications in the electrification of high‐temperature chemical synthesis. To explore this potential, model flow cells containing ≈5 wt.% Ni nanoparticles were produced through our in situ loading method, and their Joule heating performance was evaluated; the corresponding relationship between input power and the resulting temperature is displayed in Figure 6b, where a temperature of 575 °C was reached with only 20 W, demonstrating high efficiency. The electrical resistance of the Ni/PP‐CF slightly decreased from 1.75 to 1.5 Ω with increasing temperature from 400 to 575 °C for the investigated dimension size (Figure S14, Supporting Information). The resistance of the Ni/PP‐CF is slightly lower than that of PP‐CF reference, indicating that the presence of Ni nanoparticles increased the electrical conductivity. To evaluate the catalytic potential of these materials, the 3D‐printed carbon/Ni flow cells were positioned within a model reactor, as schematically illustrated in Figure 6c. They were placed between two pieces of steel wool connected to the electrodes of a power supply. Active temperature measurements were taken using a thermocouple placed within the carbon/Ni flow cell while the gaseous reaction mixture was flowed across the catalyst support. Notably, though the model flow cells used for the following catalytic study were ≈10 mm in diameter and ≈15 mm in height, our platform is scalable, and the resulting carbon‐metal oxide nanocomposites are mechanically robust. To demonstrate the capabilities of this system for scalable production, a large cylindrical specimen was prepared. This specimen was 10 cm in diameter and 5 cm in height and composed of an open‐celled grid pattern with 1.5 mm square cells and 0.6 mm walls. Upon in situ loading and pyrolysis the resulting carbon/Ni composite, shown in Figure S15a (Supporting Information), displayed shrinkage and carbon yield behaviors consistent with model samples indicating excellent scalability. Furthermore, as the mechanical integrity of supported catalysts is of particular importance for practical systems, this carbon‐Ni composite structure weighing only ≈80 g was demonstrated to support at least ≈20 kg of mass, Figure S15b (Supporting Information), indicating that these materials can be promising for practical implementation.

**Figure 7:**
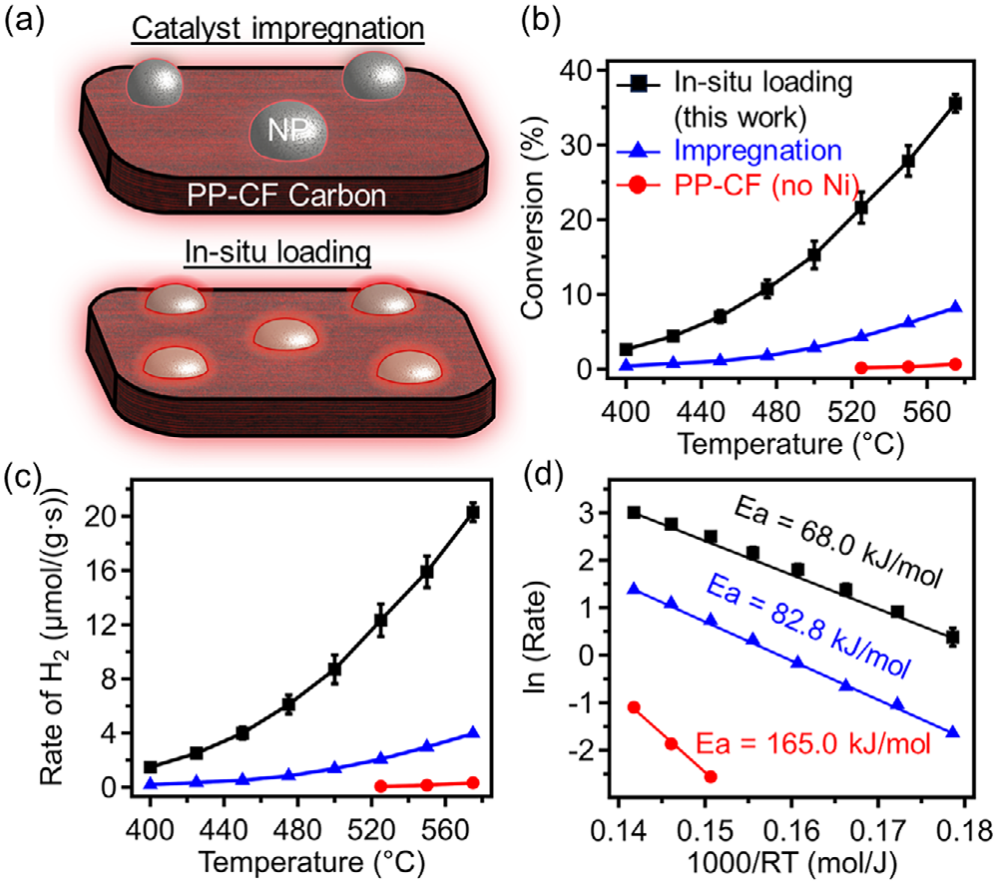
a) Schematic representation of carbon‐Ni composites produced through catalyst impregnation and in situ loading, nanoparticles (NPs) are indicated in grey (top) and pink (bottom). b) Catalytic performance of Joule heated composites assessed via NH_3_ decomposition. Black square: in situ loaded, blue triangle: catalyst impregnation, red circle: carbon control. c) Corresponding rate of H_2_ production. d) Arrhenius plots of the rate of H2 production between 3D printed carbon‐Ni nanocomposite (In situ loading), and Ni on 3D printed carbon (impregnation) counterpart. Size of the cylinder Φ10 × h15 mm (0.4±0.02 g), undiluted NH_3_ 20 mL mi^−1^n, the error bar was obtained from independent measurements of two different batches of 3D printed carbon‐Ni nanocomposite samples.

The resulting catalytic activity of the in situ loaded 3D‐printed carbon‐nickel nanocomposites for thermal catalytic NH_3_ decomposition was studied and compared to that of equivalent nanocomposites produced through the conventional impregnation method. Morphological differences between these two methods are schematically illustrated in **Figure** **7**a where our in situ loading allows for significantly enhanced contact of metal nanoparticles with the carbon catalyst support and reduced particle size in contrast to the impregnation counterpart. Figure 7b shows that our in situ loading method greatly improves the Joule heating catalytic activity in NH₃ decomposition when compared to the impregnation counterpart under the same conditions (both for Joule heating catalysis with the same Ni loading and space velocity). The in situ loaded sample demonstrated activity at temperatures as low as 400 °C, achieving ≈3% NH₃ conversion while maintaining a power output of 11.5 W. Moreover, the NH_3_ conversion increases exponentially with increasing reaction temperature, reaching ≈36% at 575 °C. In contrast to the in situ loaded nanocomposite, the carbon/Ni sample prepared via the conventional impregnation method (exposing carbon samples to the metal salt solution followed by H_2_ reduction) shows significantly lower catalytic activity in Joule heating. This result indicates that our method is more effective in promoting active catalytic sites within the carbon matrix. Additionally, the PP‐CF carbon without Ni loading is nearly inactive for NH₃ decomposition, confirming the key role of the well‐dispersed Ni nanoparticles in driving the catalytic process. Similarly, the rate of H_2_ production (Figure 7c) over the in situ loaded carbon‐nickel nanocomposite at 575 °C is ≈20 µmol/(g•s), which is more than four times that of its conventional counterpart prepared from the impregnation method. Table S2 (Supporting Information) shows the catalytic performance of other Ni based catalysts in conventional thermocatalytic NH_3_ decomposition. It is observed that the NH_3_ conversion and rate of H_2_ production is highly dependent on the reaction conditions. While the rate of H_2_ production from our study seems lower than the reported studies, it can be enhanced by increasing the space velocity. We also note that the reaction temperatures examined here are significantly lower than the pyrolysis temperature of crosslinked PP‐metal nitrate composites (800 °C) used for producing carbon Joule heaters. Additionally, while the stability of these carbonaceous Joule heaters may be a challenge in oxidative environments, the temperature for NH_3_ reacting with carbon is 700 °C and above.^[^ ^69^ ^]^ Therefore, our catalytic reactors should remain chemically stable during this reaction. The significant improvement in performance underscores the superiority of our in situ method for enhancing catalytic efficiency in electrified chemical synthesis.

From the Arrhenius plots of the rate of H_2_ (see Figure 7d), the apparent activation energy of Joule heating catalytic NH_3_ decomposition over the 3D printed carbon‐Ni nanocomposite is 68 kJ mol^−1^, which is much lower than that over the conventional impregnation counterpart (82.8 kJ mol^−1^). As demonstrated in Figure S16 (Supporting Information), in situ loading allows us to leverage the coordination of metal ions with sulfonated PP. Compared to traditional impregnation followed by calcination and reduction—where metal cations are merely chemisorbed on the carbon surface—our in situ approach facilitates the formation of smaller, more well‐dispersed nanoparticles upon pyrolysis. Therefore, our method results in catalytic Joule heaters with higher activity and lower activation energy in NH_3_ decomposition than the latter (conventional counterparts prepared by post‐impregnation) due to the structure sensitivity of the NH_3_ decomposition,^[^ ^70^, ^71^ ^]^ as well as the confinement effect of the carbon encapsulation. Additionally, the activation energy of Joule heating NH_3_ decomposition over the carbon control was 165 kJ mol^−1^, significantly higher than both Ni‐loaded samples prepared by in situ loading and impregnation methods, confirming that Ni nanoparticles are the key catalytically active sites for NH_3_ decomposition. According to an early study by McCabe,^[^ ^72^ ^]^ the activation energy of NH_3_ decomposition over Ni wires at temperatures below 727 °C is up to 211 ± 20 kJ mol^−1^ and the rate is independent of NH_3_ pressure. From literature examples, over supported Ni/SiO_2_ catalysts, the activation energy of NH_3_ decomposition was found to range from 108 to 131 kJ mol^−1^ according to Atsumi et al.^[^ ^73^ ^]^ Whereas, according to Zhang et al., the activation energy is between 100 and 120 kJ mol^−1^ (depending on the particle size) over the Ni/Al_2_O_3_ catalyst.^[^ ^74^ ^]^ The activation energies from the Joule heating catalytic NH_3_ decomposition in this work are significantly lower than these reported results, which can be likely due to the electric field/current effect on the catalysis.^[^ ^75^ ^]^ We suggest that the presence of electric current/field could enhance the recombinative desorption of N_2_ since the associative desorption of adsorbed nitrogen atoms has been considered as the rate‐determining step in NH_3_ decomposition,^[^ ^76^ ^]^ while similar observation of the positive influence of the electric field on the catalytic activity of NH_3_ decomposition have also been discussed in the literature.^[^ ^77^ ^]^

## 3 Conclusion

We report a transformative and modular approach to create highly efficient carbon‐based Joule heaters embedded with catalytic metal(oxide) nanoparticles utilizing a scalable platform. This system enables the generation of carbon‐based nanocomposites containing well‐dispersed metal(oxide) nanoparticles with tunable loading levels, particle size, and chemical identity through the infusion of sulfonated PP‐CF structures with metal nitrate precursor solutions prior to pyrolysis. Due to the inclusion of CFs in the precursor filaments, 3D‐printed structures resulting from our method display near‐zero shrinkage relative to their printed dimensions upon conversion from polymer to carbon. Our method enables dimensionally accurate fabrication of carbon‐metal oxide nanocomposite Joule heaters with robust mechanical properties. Importantly, our in situ metal precursor loading method yields structures that exhibit significantly reduced activation energies for H₂ production via NH_3_ decomposition compared to conventional methods of incorporating metal nanoparticles on carbon scaffolds (e.g., post‐impregnation method), while maintaining very low power consumption in reactions. Our approach enhances both the efficiency and sustainability of Joule heating catalytic reactions, making it highly suitable for energy‐efficient applications in diverse chemical reactions. This work demonstrates a key advancement in electrifying chemical synthesis and reducing energy requirements with the goal of assisting in decarbonization efforts within the chemical and energy industries.

## 4 Experimental Section

### Materials Preparation

3D printed precursors were prepared through fused filament fabrication (FFF) printing of commercial Braskem FL900 polypropylene filament containing ≈15 wt.% CF (PP‐CF) purchased from Dynamism. Printing was performed using an Ultimaker S3 3D printer with an Ultimaker CC 0.6 mm print core. Key FFF printing parameters in our experiments include: a nozzle temperature of 250 °C, a print bed temperature of 80 °C with Magigoo PP bed adhesive, a layer height of 0.2 mm, and a print speed of 40 mm ^−1^s. Model precursor systems used throughout this study were 1.5 cm cubic specimens with no top, bottom, or wall layers, leaving a structure purely composed of a gyroid infill pattern with a 20% infill density. The mass of these samples after printing, sulfonation, and carbonization were determined using an electronic balance.

In a typical sulfonation process, a printed sample was placed in a beaker and submerged in 98% wt.% sulfuric acid purchased from Fisher scientific. These beakers were then placed in a Thermo Scientific Thermolyne F6010 muffle furnace under air at 150 °C for various periods of time to allow the cross‐linking reaction to occur. Once removed samples were passively cooled to room temperature and washed at least three times using deionized (DI) water, which was obtained from passing tap water through a Milli‐Q IQ 7003 ultrapure lab water purification system from Millipore Sigma. Neutralization of acid waste was confirmed using pH paper and the sulfonated samples were allowed to dry under ambient conditions for 24 h. Subsequently, crosslinked PP‐CF parts were submerged and heated (at 40 °C for 16 h) in solutions of different metal nitrate‐hexahydrate/hemipentahydrate salts (including copper(II) nitrate hemipentahydrate, nickle(II) nitrate hexahydrate, magnesium(II) nitrate hexahydrate and zinc(II) nitrate hexahydrate, all purchased from Sigma–Aldrich), containing 50:50 volumetric mixture of water and ethanol. This step was necessary to introduce metal (oxide) precursors into the hydrophilic polymer scaffolds. The molar concentration of metal nitrate salt was varied between 0.25 and 1 m to achieve a range of nanoparticle loading levels in final carbonized parts. After soaking for the elapsed time, these samples were removed and allowed to dry under ambient conditions for 24 h. These samples then underwent a carbonization procedure in an Across International TF1400 tube furnace under a nitrogen atmosphere at 1 °C min^−1^ from room temperature to 600 °C, and thereafter at 5 to 800 °C, which was held for 3 h.

A control sample of nickel nanoparticle impregnated into 3D carbon was prepared through incipient wetness impregnation with Ni(II) nitrate hexahydrate (Ni (NO_3_)_2_·6H_2_O (Acros Organics)) as the precursor. Specifically, the required amounts of Ni (NO_3_)_2_·6H_2_O solution (≈0.4 mL) were added dropwise into the 3D carbon (pre‐dried at 100 °C for 12 h) to achieve a nominal Ni loading of 5 wt.%. The sample was impregnated for 24 h under ambient conditions and then dried at 100 °C for 12 h. The obtained sample was finally reduced under 10% H_2_/Ar at 550 °C for 2 h prior to performing the Joule heating catalytic NH_3_ decomposition.

### Sample Characterization Methods

Differential scanning calorimetry (DSC) was performed using a TA Instruments Discovery 250 DSC. A heat‐cool‐heat cycle was employed with an initial heating cycle from 20 to 220 °C at a rate of 10 °C min^−1^ to erase thermal history. Samples were cooled to 20 °C at a rate of 5 °C min^−1^ and then heated back to 220 °C at a rate of 10 °C min^−1^. Data analysis was performed using Trios software. The degree of crystallinity was determined by comparing the measured enthalpy of melting events to that of a theoretical value from 100% crystalline PP polymer, which was 209 J g^−1^. The carbon yield of samples was determined by comparing the mass after carbonization to the initial mass of the sample. A PerkinElmer Frontier attenuated total reflection Fourier transform infrared (FTIR) spectrometer was used to monitor changes in the chemical composition of sulfonated parts as a function of reaction time. The scan range was 4000–600 cm^−1^ with 32 scans and a resolution of 4 cm^−1^. The metal loading level corresponding to each solution molarity was assessed through thermogravimetric analysis (TGA) using a Discovery 550 TGA with platinum high‐temperature pans. 10–15 mg of carbonized samples were heated at a rate of 10 °C min^−1^ to 800 °C under air and held for 1 h to ensure a complete thermal degradation of the carbon matrix. Dimensional change upon carbonization was assessed by comparing the initial critical dimensions of a printed part (e.g., length, width, height) to those same dimensions in the final carbon‐based nanocomposite.

A liquid nitrogen physisorption experiment of PP‐CF‐derived carbon composites was conducted using a Tristar II 3020 surface area and pore size analyzer (Micromeritics). Pore size distribution and micropore volume was calculated from the sorption isotherms using the Barrett–Joyner–Halenda (BJH) model and t‐plot method, respectively, while surface area was determined through Brunauer–Emmett–Teller (BET) analysis. A Zeiss Ultra 60 field‐emission scanning electron microscopy (SEM) was used to understand morphological changes in the printed structures after sulfonation for various amounts of time and after the carbonization process using an in‐lens detector with an accelerating voltage of 10 kV. Electron dispersive X‐ray spectroscopy (EDX) was performed using a Thermoscientific UltraDry EDS spectrometer using a backscatter detector with an aperture size of 120 µm and an accelerating voltage of 10 kV, and data was analyzed using NSS software.

X‐ray photoelectron spectroscopy (XPS) analysis was performed using a Thermo‐Fisher ESCALAB Xi+ spectrometer equipped with a monochromatic Al X‐ray source (1486.6 eV, 400 µm diameter spot size). Measurements were performed using the standard magnetic lens mode and charge compensation. The base pressure in the analysis chamber during spectral acquisition was ≈4 × 10^−7^ mBar. Spectra were collected at a takeoff angle of 90° from the plane of the surface. The pass energy of the analyzer was set at 20 eV for high‐resolution scans and 150 eV for survey scans, with energy resolutions of 0.1 and 1.0 eV, respectively. Binding energies were calibrated with respect to *C 1s* at 285.3eV. All spectra were recorded using the Thermo Scientific Avantage software. Transmission electron microscopy (TEM) images were obtained using a JEOL 2100TEM (accelerating voltage 200 kV) equipped with a Gatan camera. Average particle sizes were calculated based on 30 individual measurements taken via ImageJ. Moreover, particle size analysis was performed using Image J software. Thirty nanoparticles were measured for each given concentration. X‐ray powder diffraction (XRD) patterns were collected on a Rigaku Ultima III X‐ray diffractometer with monochromatic Cu Kα radiation (154.06 pm, 40 kV, and 44 mA) with a scan speed of 4°/min.

### Joule Heating Enabled Catalytic NH_3_ Decomposition

The in‐house established Joule heating catalytic reactor was consisting of an ½ inch quartz tube with an inner diameter of 10 mm. The quartz reactor was wrapped with a 1 cm thickness of ceramic fiber to reduce heat loss. The carbon cylinder joule heater/catalyst with the same diameter as the quartz tube was sandwiched between two conductive steel wool and loaded at the center of the quartz reactor. A 1/16 inch copper rod and a K‐type thermocouple were used as electrodes in contact with the steel wool. The tip of the thermocouple was inserted into the carbon‐nanoparticle samples for the bulk temperature measurement during the reaction (or under inert to measure the Joule heating performance). The electrodes from both sides of the reactor were connected to an Eventek DC Power Supply (30V/10A Variable Power Supply). The temperature of the catalyst was online monitored with a Platinum series universal benchtop temperature controller (Omega Engineering). The catalyst was in situ activated at 550 °C for 30 min under 10% H_2_/Ar (20 mL mi^−1^n). After activation, the temperature was tuned to below 200 °C by decreasing the current passed through the carbon cylinder, and the reactor inlet was switched to undiluted NH_3_ (at different flow rates depending on the desired space velocity). Then the by‐pass NH_3_ signal was measured by an online mass spectrometer (Leybold Inficon Transpector Residual Gas Analyzer TSP TH100). After the NH_3_ signal (m/z = 16) became stable, the temperature of the carbon cylinder was tuned to 400–550 °C (by increasing the current) for NH_3_ decomposition. The temperature was kept for at least 15 min until the reaction reached a steady‐state (the signals of m/z = 16 and m/z = 28 became stable). The MS signal intensities of NH_3_ and N_2_ were converted to partial pressure based on external standard calibration (partial pressure/intensity relationship). Finally, the mole flow rate of NH_3_ and N_2_ were calculated based on the ideal gas equation of state (P_i_v = F_i_RT), where P_i_ is the partial pressure of selected molecules (Pa), v is the volumetric flow rate (mL S^−1^) at the exit of the reactor, F_i_ is the mole flow rate (mol S^−1^), R is the ideal gas constant (J mol^−1^ K^−1^), and T is the temperature (K). NH_3_ conversion was calculated based on Equation (1).

(1) $$x=\frac{F_{NH_{3},in}-F_{NH_{3},out}}{F_{NH_{3},in}}$$

where $F_{NH_{3},in}$ and $F_{NH_{3},out}$ are the mole flow rate (mol S^−1^) of NH_3_ at the inlet and outlet of the reactor. Noteworthily, due to the change in the total number of moles in the NH_3_ decomposition, the volumetric flow rate *v* = *v_in_* (1 + *x*).

The rate of H_2_ was calculated based on the mole flow rate of H_2_ ($F_{H_{2}}=1.5\;\times \;\left(F_{NH_{3},in}-F_{NH_{3},out}\right)$).

(2) $$Rate=\frac{F_{H_{2}}}{mass\;of\;carbon\;cylinder}$$

## Conflict of Interest

The authors declare the following competing interests: Z. Q. and P. S. submitted two U.S. patent applications for relevant technology of AM of carbons (U.S. Application No. 17/848342; No. 18/112446). The remaining authors declare no competing interests.

## Supporting information



Supporting Information

## Data Availability

The data that support the findings of this study are available from the corresponding author upon reasonable request.
