# Supplementary material for: Transformative 3D Printing of Carbon‐metal Nanocomposites as Catalytic Joule Heaters for Enhanced Ammonia Decomposition
Source: Adv Sci (Weinh). 2025 Apr 27;12(20):2413149. doi: 10.1002/advs.202413149 (PMC12120704; doi:10.1002/advs.202413149)
Supplement: Supplementary file 1 — Supporting Information [file ADVS-12-2413149-s001.docx]

**Supporting Information For:**

**Transformative 3D Printing of Carbon-metal Nanocomposites as Catalytic Joule Heaters for Enhanced Ammonia Decomposition**

Paul Smith^a^, Jiachun Wu^b^, Anthony Griffin^a^, Kaleb Jones^a^, Jeff Aguinaga^a^, Ethan Bounds^a^, Derek Patton^a^, Yizhi Xiang^b,c,*^, Zhe Qiang^a,*^

^a^School of Polymer Science and Engineering, University of Southern Mississippi, Hattiesburg, MS, 39406, USA

^b^Dave C. Swalm School of Chemical Engineering, Mississippi State University, Mississippi State, Mississippi 39762, USA

^c^Current address: Department of Chemical and Biomedical Engineering, University of Missouri, Columbia, Missouri 65211, USA

Corresponding authors: Y. X. (yxpxb@missouri.edu) and Z. Q. (zhe.qiang@usm.edu)


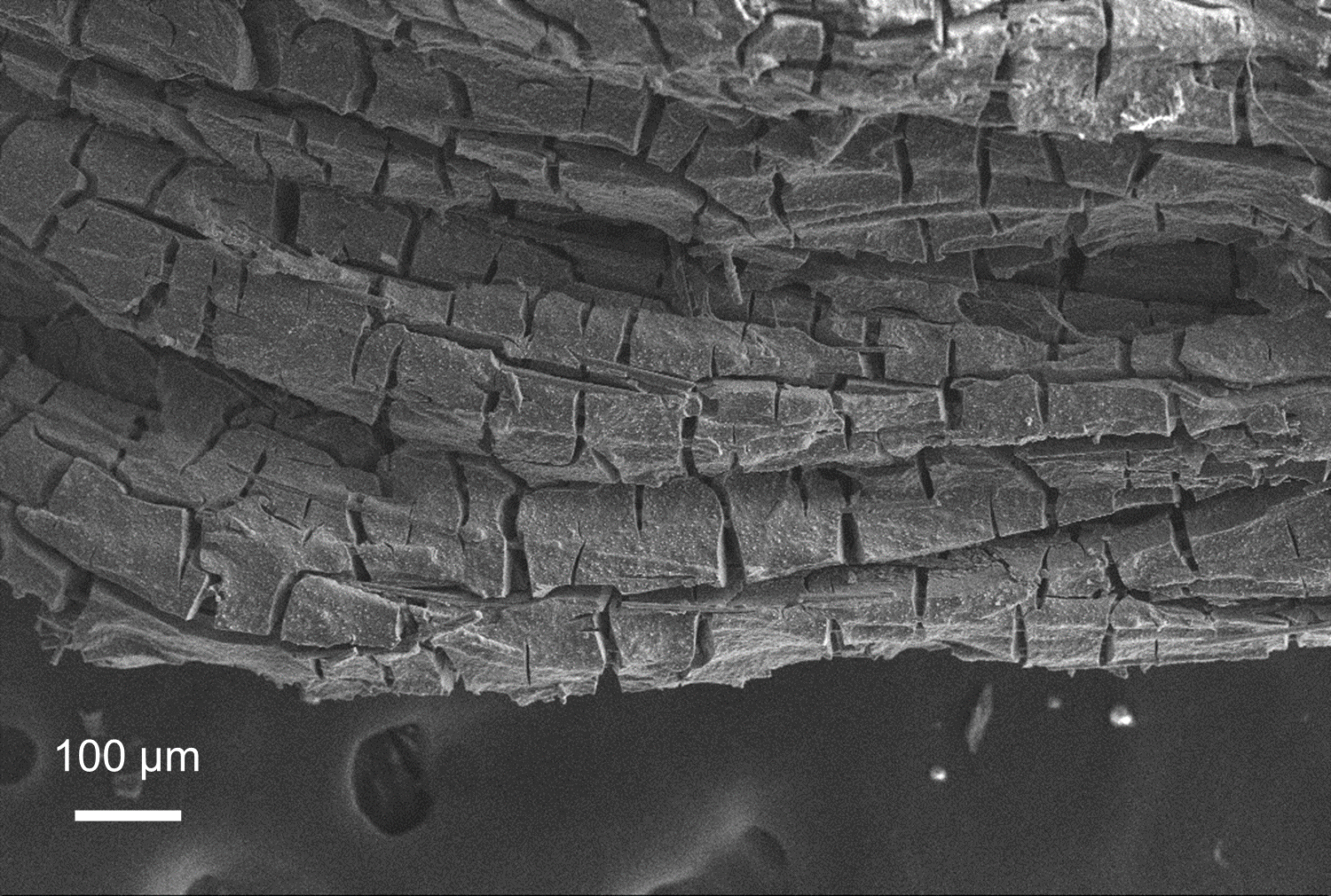


Figure S1. The formation of micro-cracks during the sulfonation of PP-CF at 150 °C after 12 h of reaction time.


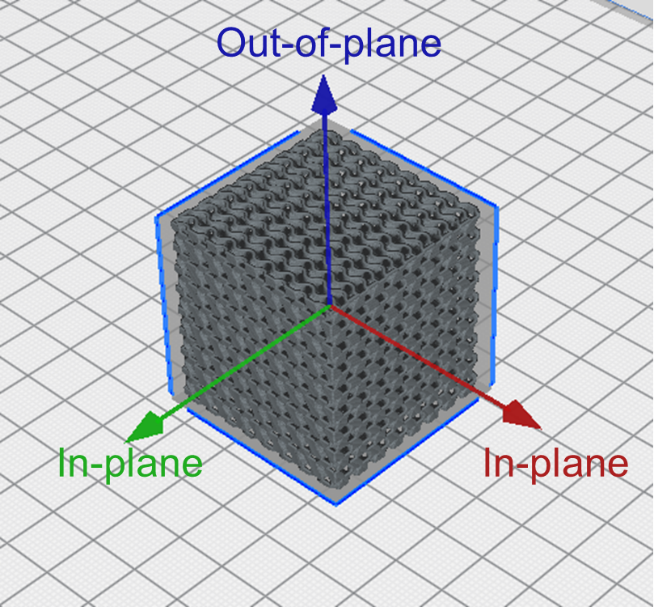


Figure S2. Conventions for the directions of 3D printed parts.


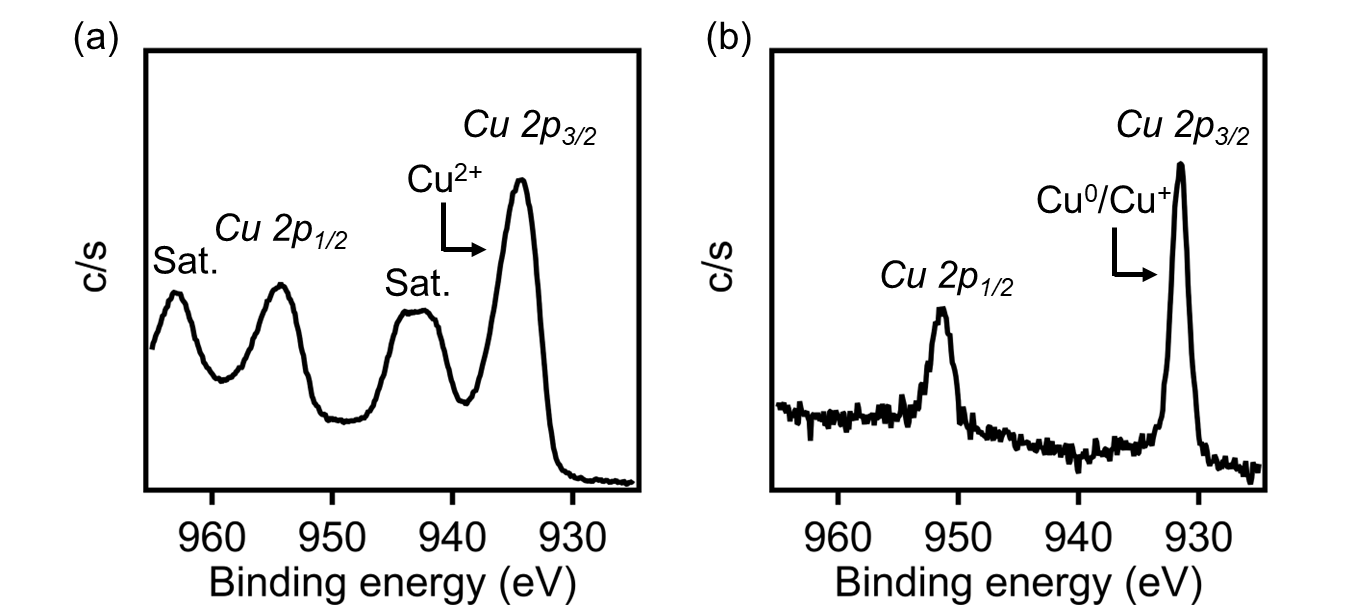


Figure S3. (a) Cu2p spectra of copper(II) nitrate-hemipentahydrate salt indicating the presence of a Cu^2+^ species. (b) Cu2p spectra of sulfonated PP-CF sample treated with a copper solution indicating the presence of an elemental or Cu^+^ state; this sample is not carbonized.


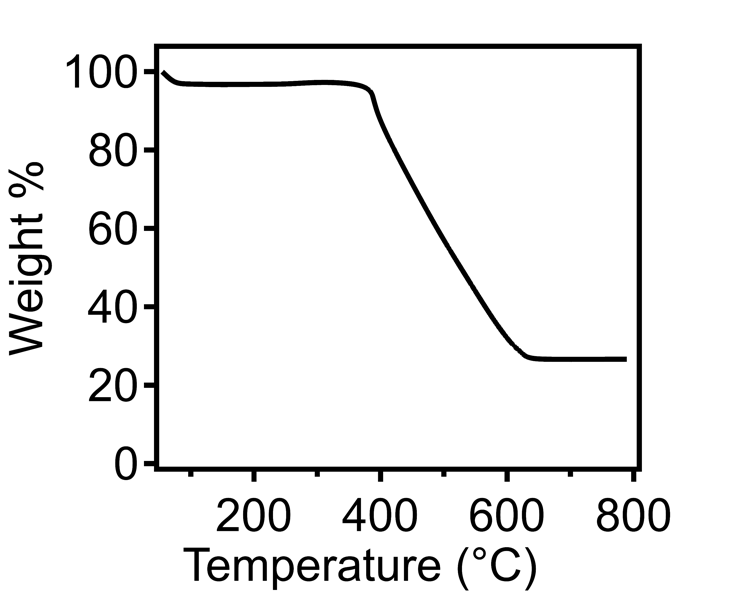


Figure S4. Representative TGA curve of PP-CF-derived carbons, treated with 1 M copper(II) nitrate-hemipentahydrate solution prior to pyrolysis, under air.


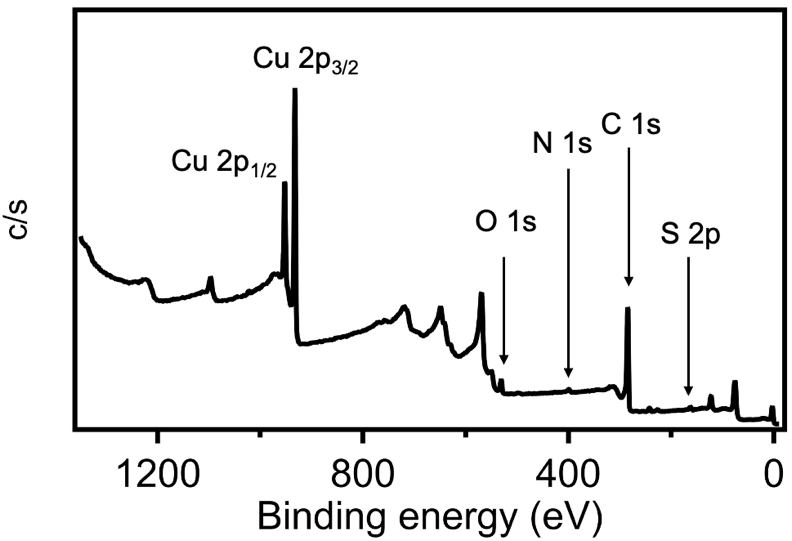


Figure S5. XPS survey scan of PP-CF derived carbon treated with 1 M copper nitrate hemipentahydrate solution. Elemental composition was determined to be carbon (~80 at%), copper (~10 at%), oxygen (~5 at%), and sulfur (~1.3 at%).


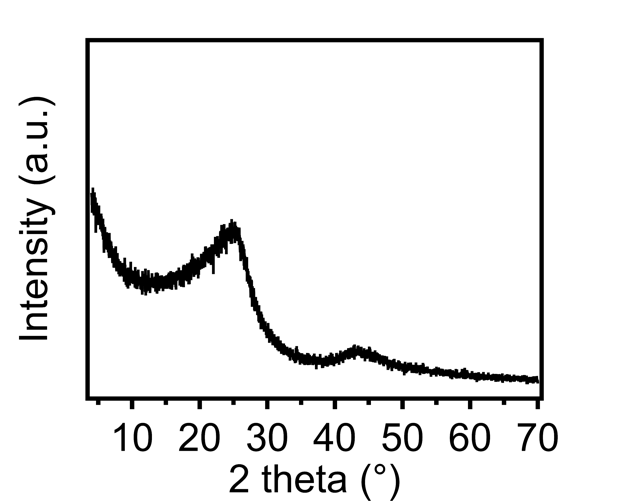


Figure S6. XRD spectra of PP-CF derived carbon crosslinked for 12 h without metal treatment.


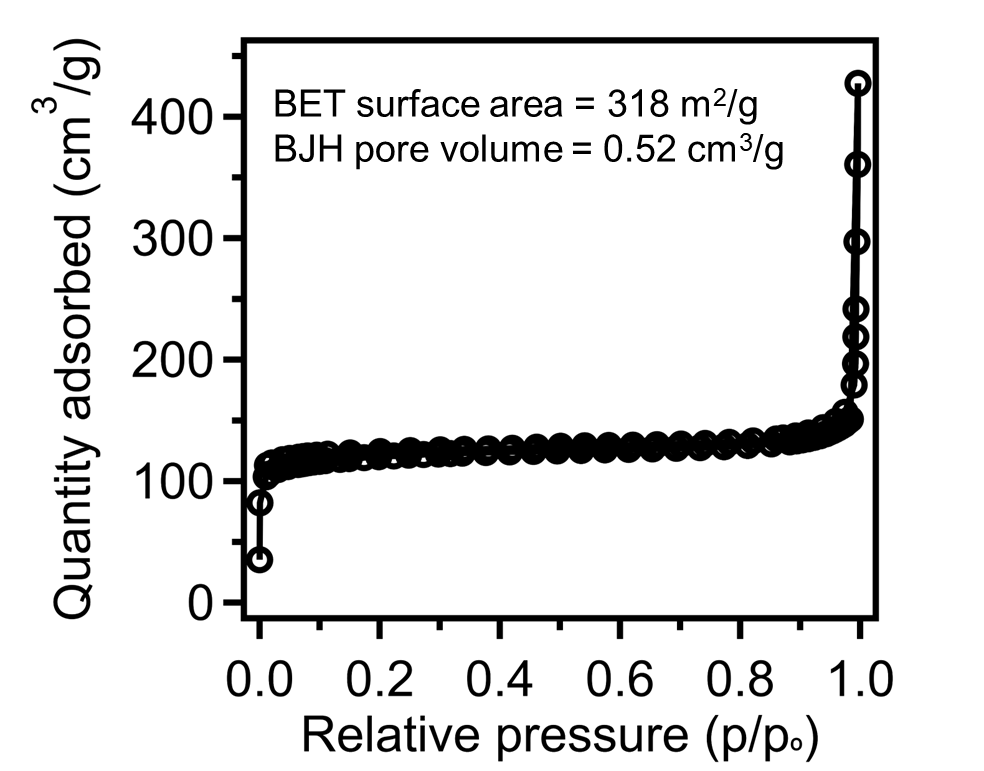


Figure S7. Representative nitrogen physisorption isotherm of PP-CF derived carbon crosslinked for 12 h without metal inclusion.


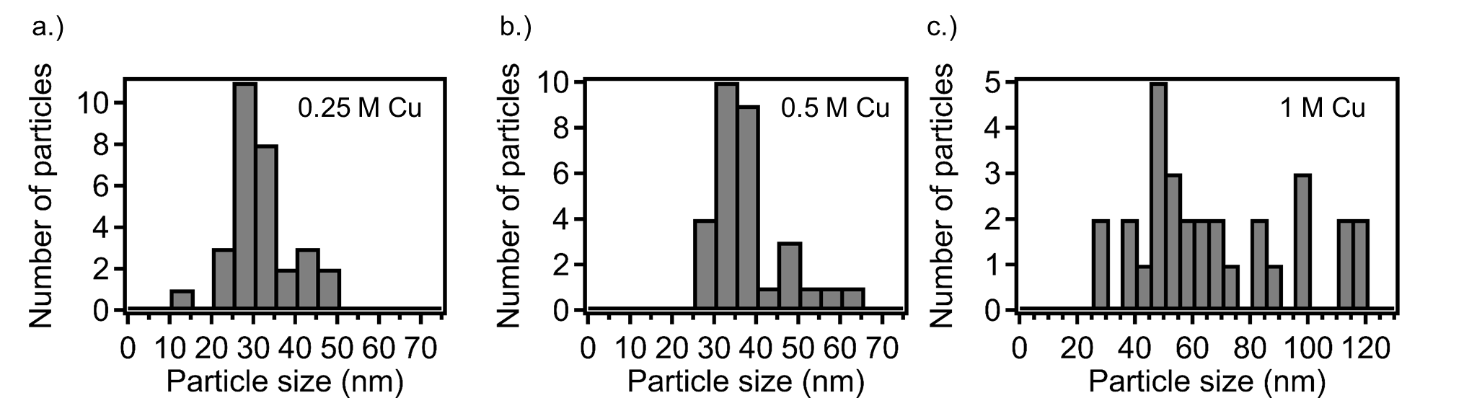


Figure S8. Particle size distributions from TEM of PP-CF/Cu composite materials as a function of copper(II) nitrate hemipentahydrate solution concentration for n=30 measured particles. Number of particles is presented on the y-axis and the total number is distributed into bin sizes of 5 nm.


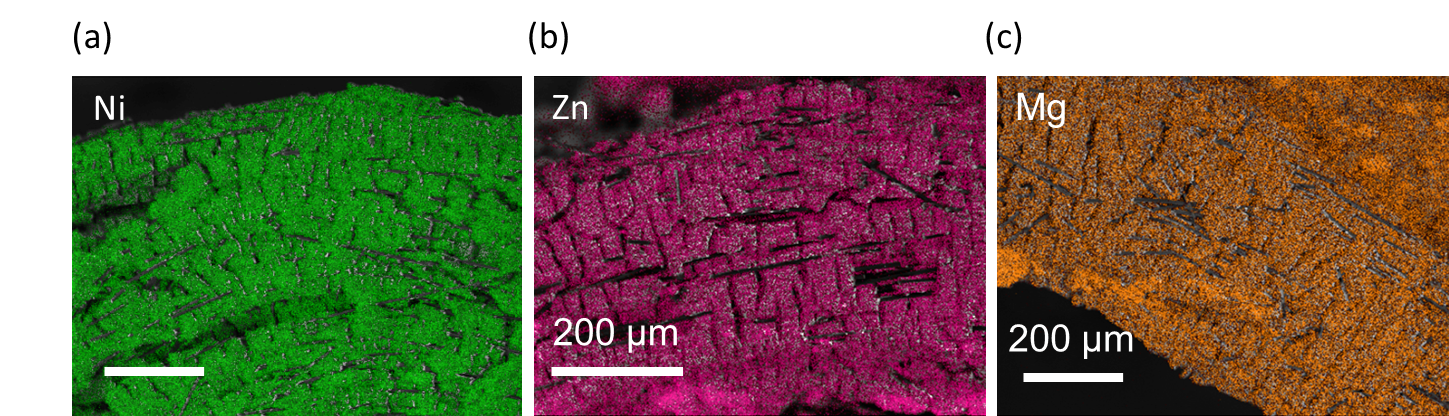


Figure S9. EDX spectral mapping of PP-CF derived carbons treated with (a) 1M nickel solution (b) 1 M zinc solution and (c) 1M magnesium solution prior to pyrolysis.


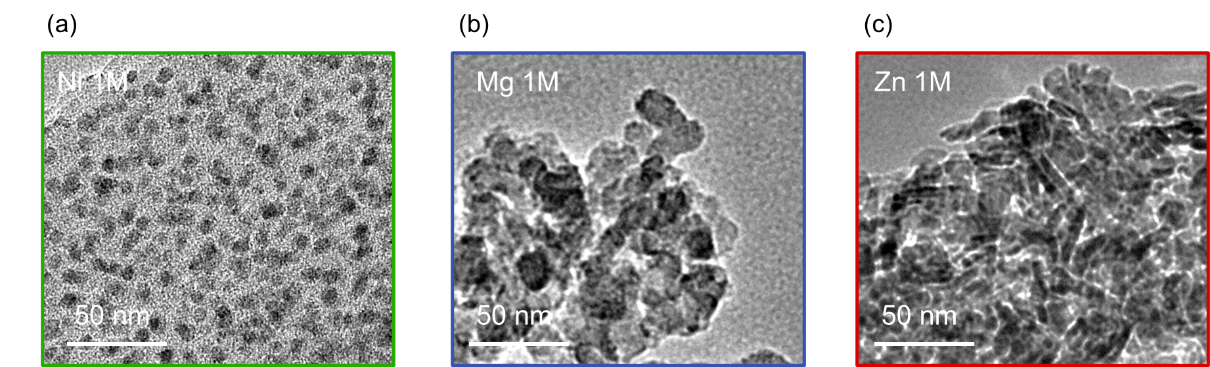


Figure S10. TEM images of carbon-metal nanocomposites derived from 1 M metal nitrate solutions of: (a) nickel (Ni), (b) magnesium (Mg), and (c) zinc (Zn).


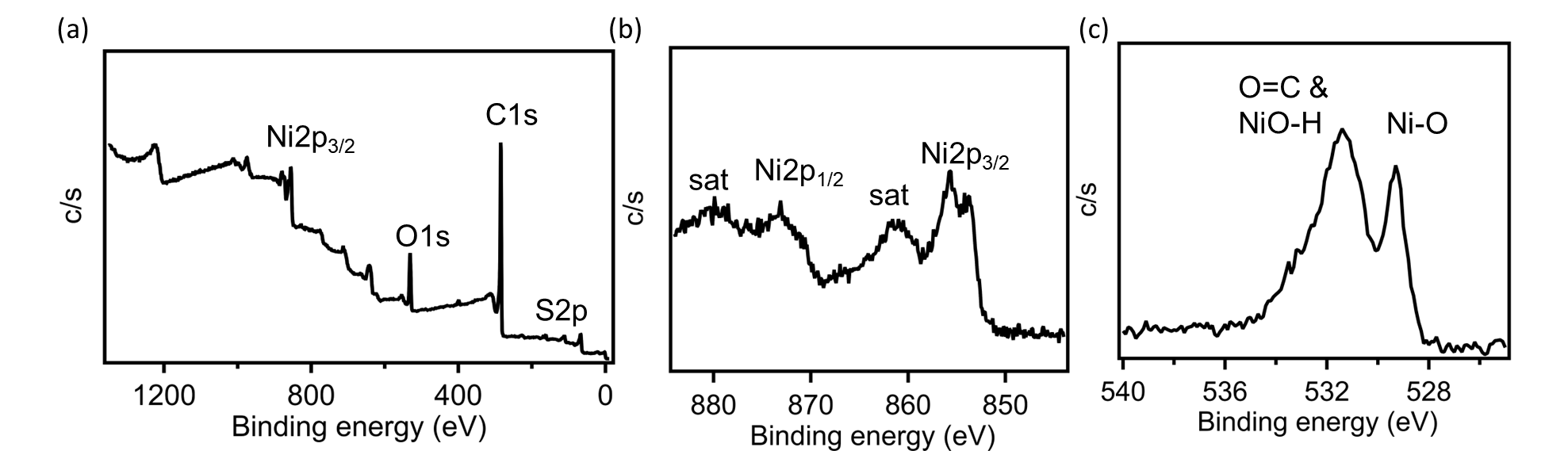


Figure S11. XPS scans of PP-CF carbon treated with 1 M nickel solution prior to pyrolysis (a) XPS survey scan (b) Ni2p high resolution scan where *Ni 2p_3/2_* peaks 853.7 and 855.6 eV, corresponding to the contributions of NiO and Ni(OH)_2_ (c) O1s high resolution scan.


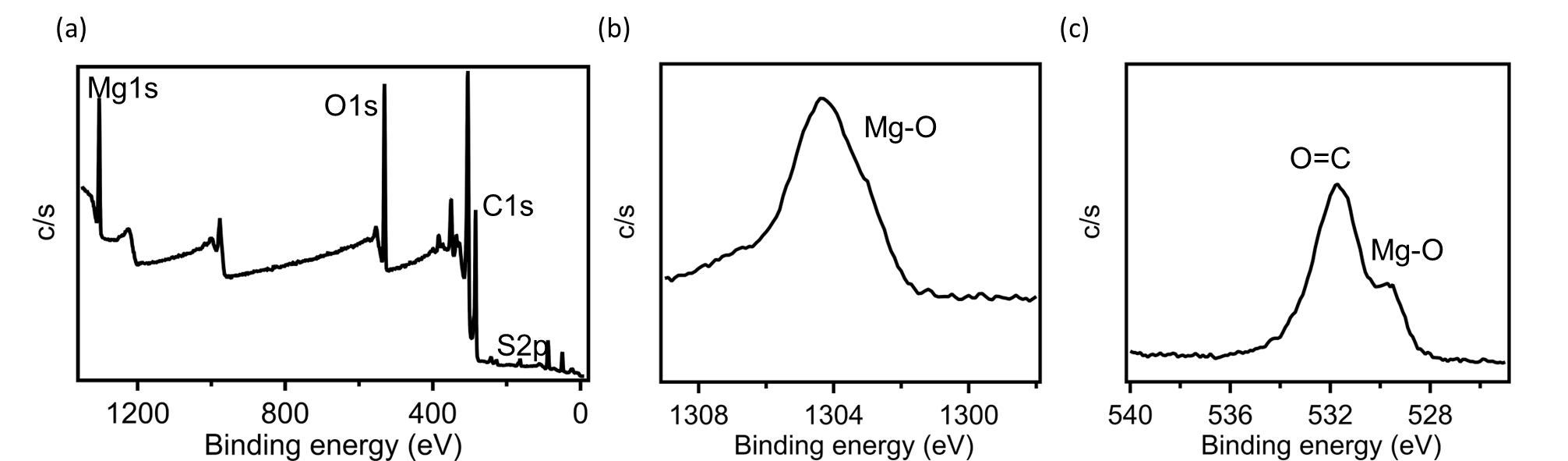


Figure S12. XPS scans of PP-CF carbon treated with 1 M magnesium solution prior to pyrolysis (a) XPS survey scan (b) Mg1s high resolution scan (c) O1s high resolution scan.


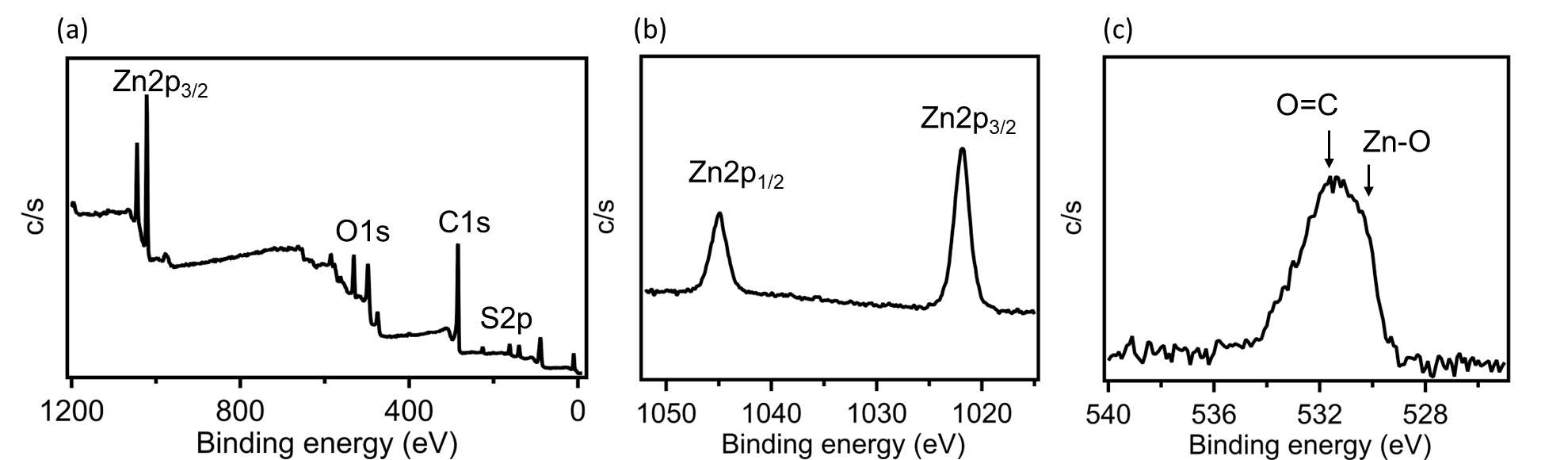


Figure S13. XPS scans of PP-CF carbon treated with 1 M zinc solution prior to pyrolysis (a) XPS survey scan (b) Zn2p high resolution scan (c) O1s high resolution scan.





Figure S14. Electrical resistivity of the Ni/PP-CF and PP-CF reference calculated under the reaction conditions.


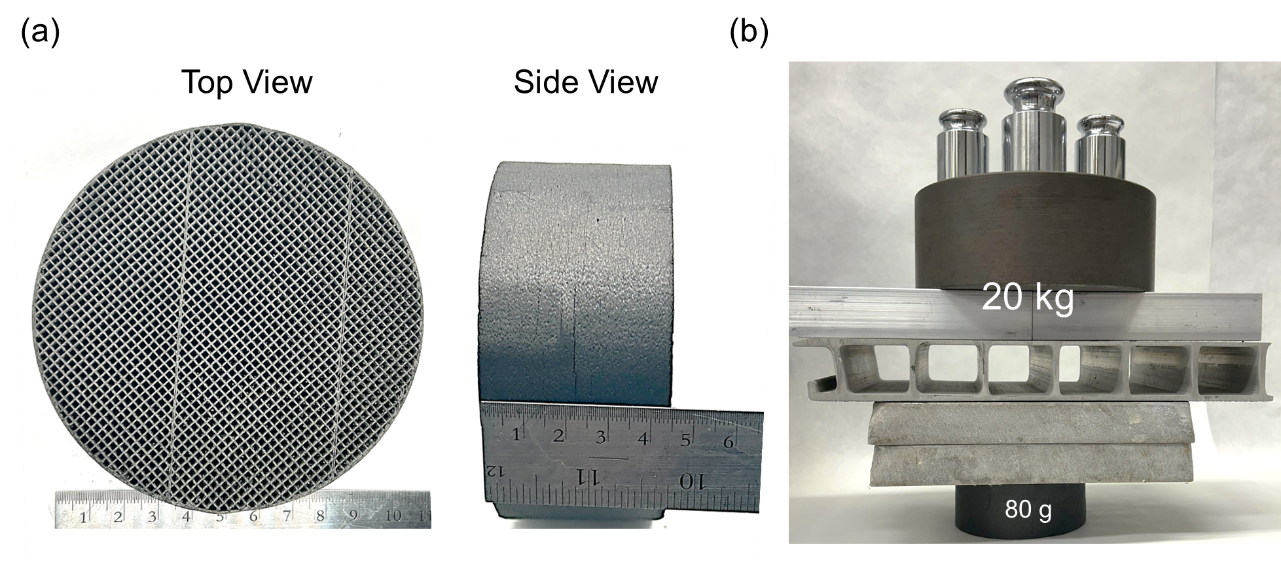


Figure S15. (a) Picture of carbon/Ni flow cell demonstrator printed with 1.5 mm grid geometry and 0.6 mm walls. The initial diameter of this structure was 10 cm in diameter and 5 cm in height, structural retention and carbon yield were consistent with model specimens. (b) The same demonstrator structure (bottom) with a mass of 80 g supporting at least 20 kg of metal weights indicating the robust mechanical properties of these materials.


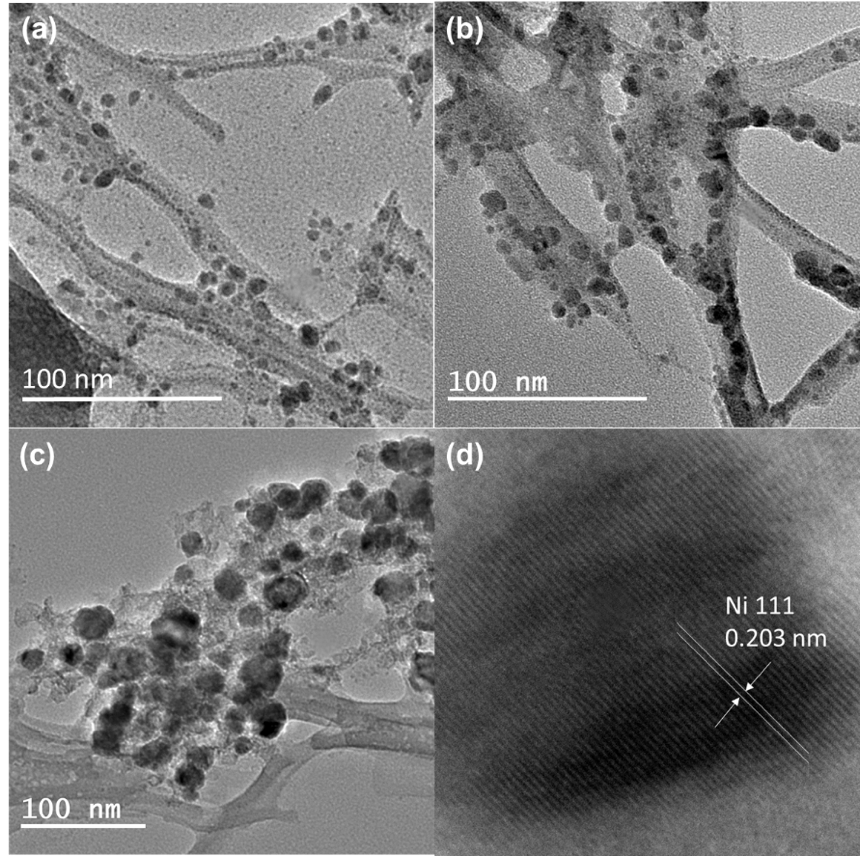


Figure S16. (a) and (b), TEM images of fresh (panel (a)) and used (panel (b) 3D printed carbon-Ni nanoparticle composite produced through the in-situ method, (c) TEM image of used Ni/3D carbon produced from the impregnation method, (d) high resolution TEM of selected Ni nanoparticle.

Table S1. Masses of model PP-CF/copper(II) nitrate hemipentahydrate system throughout the in-situ loading process reflecting the effects of copper loading on the bulk mass of these samples.

| Solution concentration (M) | Printed mass (g) | Sulfonated mass (g) | Pyrolyzed mass (g) |
| --- | --- | --- | --- |
| 0.25 | 0.924 | 1.343 | 0.621 |
| 0.5 | 0.910 | 1.312 | 0.637 |
| 1 | 0.919 | 1.331 | 0.683 |

Table S2. Catalytic performance of Ni based catalyst in thermocatalytic NH_3_ decomposition reported in literatures.

| Catalyst | Ni loading (%) | T (℃) | GHSV (mL/(h·g_cat_)) | Conversion (NH_3_) | H_2_ rate (mmol/(min·g_cat_)) | Ref. |
| --- | --- | --- | --- | --- | --- | --- |
| Ni/C | 5 | 575 | 3,000 | 36 | 1.2 | This work |
| Ni/La_2_O_3_ | 26.5 | 550 | 6,000 | 74.1% | 5.0 | 1 |
| Ni/AC | 5.0 | 500 | 6,000 | 24.8% | 1.7 | 2 |
| Ni/MWCNT | 10.0 | 500 | 6,000 | 57.6% | 3.9 | 2 |
| Ni/AC | 10.0 | 700 | 30,000 | 40.9% | 13.7 | 3 |
| Ni/ZSM-5 | 5.0 | 650 | 30,000 | 50.1% | 16.8 | 4 |
| Ni/TiO_2_ | 40.0 | 550 | 6,000 | 30.0% | 2 | 5 |

References:

[1] H. Muroyama, C. Saburi, T. Matsui, K. Eguchi, *Appl. Catal. A. Gen.* **2012**, *443–444*, 119.

[2] H. Zhang, Y. A. Alhamed, Y. Kojima, A. A. Al-Zahrani, H. Miyaoka, L. A. Petrov, *Int. J. Hydrogen Energy* **2014**, *39*, 277.

[3] T. Meng, Q.-Q. Xu, Y.-T. Li, J.-L. Chang, T.-Z. Ren, Z.-Y. Yuan, *J. Ind. Eng. Chem.* **2015**, *32*, 373.

[4] Z.-P. Hu, C.-C. Weng, C. Chen, Z.-Y. Yuan, *Appl. Catal. A. Gen.* **2018**, *562*, 49.

[5] K. Okura, K. Miyazaki, H. Muroyama, T. Matsui, K. Eguchi, *RSC Adv.* **2018**, *8*, 32102.
